# Glucocorticoid receptor coordinates transcription factor-dominated regulatory network in macrophages

**DOI:** 10.1186/1471-2164-15-656

**Published:** 2014-08-06

**Authors:** Yurii Chinenov, Maddalena Coppo, Rebecca Gupte, Maria A Sacta, Inez Rogatsky

**Affiliations:** Hospital for Special Surgery, The David Rosensweig Genomics Center, 535 East 70th Street, New York, NY 10021 USA; Graduate Program in Biochemistry, Cell and Molecular Biology, Weill Cornell Graduate School of Medical Sciences, 1300 York Avenue, New York, NY 10021 USA; Weill Cornell/Rockefeller/Sloan-Kettering Tri-Institutional MD-PhD Program, 1300 York Avenue, New York, NY 10021 USA; Graduate Program in Immunology and Microbial Pathogenesis, Weill Cornell Graduate School of Medical Sciences, 1300 York Avenue, New York, NY 10021 USA

**Keywords:** Transcriptional regulation, Glucocorticoid receptor, Inflammation, Feed forward loops, Gene regulatory network, KLF transcription factors

## Abstract

**Background:**

Inflammation triggered by infection or injury is tightly controlled by glucocorticoid hormones which signal via a dedicated transcription factor, the Glucocorticoid Receptor (GR), to regulate hundreds of genes. However, the hierarchy of transcriptional responses to GR activation and the molecular basis of their oftentimes non-linear dynamics are not understood.

**Results:**

We investigated early glucocorticoid-driven transcriptional events in macrophages, a cell type highly responsive to both pro- and anti-inflammatory stimuli. Using whole transcriptome analyses in resting and acutely lipopolysaccharide (LPS)-stimulated macrophages, we show that early GR target genes form dense networks with the majority of control nodes represented by transcription factors. The expression dynamics of several glucocorticoid-responsive genes are consistent with feed forward loops (FFL) and coincide with rapid GR recruitment. Notably, GR binding sites in genes encoding members of the KLF transcription factor family colocalize with KLF binding sites. Moreover, our gene expression, transcription factor binding and computational data are consistent with the existence of the GR-KLF9-KLF2 incoherent FFL. Analysis of LPS-downregulated genes revealed striking enrichment in multimerized Zn-fingers- and KRAB domain-containing proteins known to bind nucleic acids and repress transcription by propagating heterochromatin. This raises an intriguing possibility that an increase in chromatin accessibility in inflammatory macrophages results from broad downregulation of negative chromatin remodelers.

**Conclusions:**

Pro- and anti-inflammatory stimuli alter the expression of a vast array of transcription factors and chromatin remodelers. By regulating multiple transcription factors, which propagate the initial hormonal signal, GR acts as a coordinating hub in anti-inflammatory responses. As several KLFs promote the anti-inflammatory program in macrophages, we propose that GR and KLFs functionally cooperate to curb inflammation.

**Electronic supplementary material:**

The online version of this article (doi:10.1186/1471-2164-15-656) contains supplementary material, which is available to authorized users.

## Background

Preserving homeostasis is the primary function of the innate immune system that detects “danger and stranger” signals and eliminates invading microorganisms, responds to irritation and injury and eventually initiates tissue repair. Innate immune cells, such as macrophages, neutrophils and dendritic cells constantly sample their environment for lipopolysaccharides (LPS), double and single-stranded nucleic acids, microbial proteins and other broad molecular patterns that are not normally present in eukaryotes and, in response, produce cytokines and chemokines that attract additional immune cells to the site of infection or injury. Normally a protective response, excessive or persistent inflammation is associated with tissue damage and needs to be regulated. Indeed, numerous mechanisms have evolved that control inflammation at multiple levels. Systemically, inflammatory stimuli activate neuro-endocrine circuitry that triggers the production of glucocorticoids by the adrenal glands, which ultimately attenuate the expression of inflammatory cytokines [1]. These potent anti-inflammatory properties made glucocorticoids exceedingly common for managing a wide range of autoimmune and inflammatory conditions such as rheumatoid arthritis, systemic lupus erythematosus, inflammatory bowel disease, psoriasis and multiple sclerosis [2].

Glucocorticoids signal through the glucocorticoid receptor (GR) – a ubiquitously expressed ligand-dependent transcription factor (TF) of the nuclear receptor (NR) superfamily. GR regulates transcription by either binding directly to specific DNA sequences known as glucocorticoid response elements (GREs) or by tethering to other DNA-bound regulators, such as Activator Protein (AP)1, Nuclear Factor (NF) κB and Signal Transducers and Activators of Transcription (STAT) family members [3]. Although GR is expressed in all immune cells, the physiological outcomes of GR activation are highly cell type-specific: for example, glucocorticoids are anti-apoptotic in neutrophils, but pro-apoptotic in eosinophils, dendritic and some T-cells [4]. Moreover, prolonged glucocorticoid treatment induces cells polarization toward a new phenotype with either pro- or anti-inflammatory properties [5, 6].

The analyses of glucocorticoid-regulated transcriptomes paint a complicated picture encompassing hundreds of up- and down-regulated genes that vary in different cell types and populations and over time of glucocorticoid exposure [7, 8]. Although shared GR target genes certainly exist, system-specific regulators and pathways drastically affect transcriptional outcomes, response dynamics and relative activities of such shared genes and their products. The existence of intricate inter-protein and inter-pathway interactions contributing to the NR-mediated gene regulation has been proposed almost 30 years ago [9]. The structural analysis of NR transcriptional networks, however, was lagging due to the lack of genome-wide data and limited availability of analytical tools. More recently, studies in bacteria and yeast have defined specific patterns of functional interactions (“network motifs”) between interdependent TFs and provided a computational framework for the analysis of gene expression data to identify such motifs [10–12]. The simplest motif - a positive or negative auto-regulatory loop - consists of a single TF that regulates its own expression. More complicated feed forward loops (FFL) involve three factors: a signal-responsive master regulator TF, an intermediate TF controlled by the master TF and a jointly regulated gene under the control of both the master and intermediate TFs [12]. Depending on the specific activities of the master and intermediate TFs (activation or repression) and the response thresholds of participating genes, the dynamics of the FFL transcriptional outputs vary, yielding unique expression patterns for various TF and target gene combinations. Dynamic responses elicited by FFLs deviate from simple gene regulation providing for unusual control mechanisms that are responsible for noise filtering, fold-change sensing, pulse generation and transcription response acceleration [11, 13, 14].

Gene expression networks can be represented as graphs with TFs and other expression regulators acting as nodes and functional interactions between regulators and between regulators and their targets as directional edges [12, 15]. Of particular interest are the networks and network motifs in which TFs regulate each other, or themselves act as transcriptome organizers and ultimately determine the topology of the entire network [12]. Several computational algorithms and experimental approaches have been successfully applied to map global transcriptional networks and identify novel functional motifs in various organisms [16, 17]. In metazoans, this analysis is often complicated by the lack of information about the edge identity (not all targets for a given TF are known, some known “targets” are not regulated directly) and direction (a TF can either activate or repress the same gene in a tissue-specific manner). To complicate matters further, the role of intermediate TFs can be fulfilled by miRNAs or regulators of RNA translation and stability [18, 19]. Thus, dissecting regulatory networks requires a combination of computational and experimental approaches.

We reasoned that a highly branched response to glucocorticoids is determined by the early transcriptional events. Here, we focused on the regulatory network elicited by an acute stimulation of mouse macrophages with glucocorticoids and/or LPS. Combined with high-resolution kinetic experiments and dynamics modeling, this analysis enabled us to dissect early post-stimulation events prior to extensive signal propagation, which usually masks the *bona fide* response to GR activation by a web of secondary effects.

## Results

### Transcriptome analysis of mouse macrophages exposed to acute glucocorticoid and LPS stimulation

To analyze early regulatory events initiated by glucocorticoids and inflammatory stimuli we treated BMMФ with either ethanol vehicle (U), LPS (L), Dex (D), or a combination of the two (L + D) for 1 h, isolated and sequenced PolyA-enriched RNA as described in (Additional file 1). The sequencing results are summarized in Additional file 2: Table S1.

**Figure 1:**
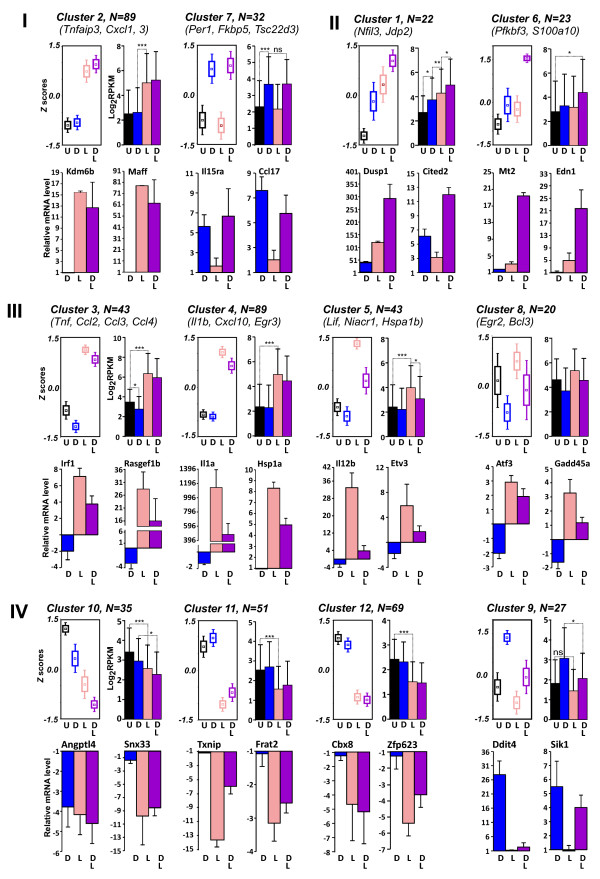
**Dex- and LPS-regulated genes in BMMФ form distinct clusters depending on specific patterns of expression.** ANOVA-filtered RNA-seq expression values (RPKM) were Z score-transformed and subjected to k-mean clustering. For each cluster, the upper left panel shows the cluster average Z score-transformed log2 RPKM for each treatment condition where the central square represents standardized cluster mean, the rectangle is the mean +/- standard deviation (SD) and the whiskers are a 95% confidence interval; the upper right panel shows log-transformed raw cluster mean expression values. Symbols representing treatment conditions are black - untreated (U), blue - Dex-treated (D), pink - LPS-treated (L) and purple - co-treated (DL) cells. The expression of two representative genes per cluster were determined by RT-qPCR with gene-specific primers and shown at the bottom. The statistical significance of differences (Mann–Whitney test) is indicated by asterisks as following: * - p < 0.05, ** - p < 0.01, *** - p < 0.001 and ns is non-significant.

To uncover the regulatory patterns in gene expression data, we performed *k*-mean cluster analysis of ANOVA-filtered differentially expressed genes (see Additional file 1). To minimize magnitude-based clustering, the log2-transformed expression values were first converted to Z-scores as in  where *X*_gene_ is an expression value for a given gene at a given condition,  is an average expression value across conditions and σ is a standard deviation of  across conditions. The optimal number of clusters was determined using the “elbow” method by plotting within-cluster variance vs. the number of clusters. The analysis was performed using Euclidian distance with progressively increasing number of clusters from 8 to 14 to determine a stable configuration using the cluster analysis module of STATISTICA 8.0. We grouped ANOVA-filtered data into 12 clusters of co-regulated genes (Additional file 2: Table S2). We further evaluated the significance of differences in gene expression within clusters by the Mann–Whitney test and validated the results for a limited number of genes by RT-qPCR. As many glucocorticoid- and LPS-responsive genes have been previously characterized by us and others [20–22], we selected for validation either poorly characterized or novel target genes. Based on patterns of co-regulation, we grouped these clusters into four larger categories (Figure 1).

#### I) Genes activated by either LPS (Cluster 2) or glucocorticoids (Cluster 7)

The majority of genes in these two clusters are upregulated by either LPS or Dex independently, with little evidence for inter-treatment interactions at 1 h. The LPS-induced *Cluster 2* (Figure 1, Mann–Whitney, P_U-L_ = 4.11*10^-13^) contains genes encoding pro- and anti-inflammatory cytokines and chemokines (Il10, Cxcl1, 3, 5 and 7, Ccl7 and Tnfsf9), TFs involved in stress response (Maff, Ets2, Fosl2 and Kdm6b) and proteins involved in TLR signaling (Tlr2, Cd14 and Cd40) and signal transduction (Itpkc, Rabgef1, Gbp5a).

*Cluster 7* contains Dex-induced genes (P_U-D_ = 0.0013), including several well-characterized GR targets such as TFs Per1 and Klf9, immunophilin Fkbp5, potassium channel Kcnk6. In addition, this cluster includes several genes whose regulation by Dex has not been previously reported: Interleukin 15 receptor alpha (Il15ra), the Wnt pathway receptor Fzd4, the TF Klf2 and chemokine Ccl17.

#### II) Genes co-activated by LPS and Dex

These genes display either predominantly additive (*cluster 1*) or synergistic (*cluster 6*) activation by LPS and Dex. Several genes in these clusters are previously characterized GR targets including Dusp1, Nfil3 and Cited2 (*cluster 1*) and Mt2 and Pfkfb3 (*cluster 6*), whereas the glucocorticoid and LPS responsiveness of several others, such as histone chaperon Jdp2 (*cluster 1*), has not been reported previously.

#### III) Genes induced by LPS and repressed by Dex

This large group of genes is represented by clusters 3, 4 and 5. *Cluster 3* contains LPS-induced genes (P_U-L_ = 1.95*10^-11^) expressed at relatively high level in resting BMMФ. The basal expression of these genes is significantly more sensitive to hormonal treatment (P_U-D_ = 0.0107) than their LPS-induced expression. This cluster encompasses a number of inflammatory cytokines (Ccl2, 3 and 4, Tnf, Tnfaip2), TFs (Ier5, Junb, Bcl6, Prdm1 and Irf1) and proteins involved in signal transduction (Gadd45b, Dusp5, Rasgef1b). Interestingly, several genes in this cluster (Ccl2, 3 and 4, Tnf) are characterized by the presence of the stalled RNA Pol II near the transcription start site in uninduced conditions and are activated primarily at the level of the Pol II pause release during early elongation [23–25]. *Cluster 4* combines a heterogeneous group of genes with low basal expression (P_Ucluster 4<Ucluster3_ = 0.0003) that are strongly induced by LPS and includes inflammatory cytokines (Il1a, Il1b, Cxcl10, Ifnb1, Tnfsf4 and Il1f9) and other direct mediators of inflammation (Ptgs2, mIR-155 host gene, Hsp1a), TFs (Egr3) and signaling molecules (Gpr84, Areg). *Cluster 5* contains many genes whose LPS induction is strongly inhibited by Dex treatment (P_L-(L+D)_ = 0.02) including several cytokines and chemokines (Il12b, Lif, Il1rn and Il17ra ) and TFs (Mxd1, Etv3, Klf7 and Ets1).

*Cluster 8* contains statistically heterogeneous previously reported (Atf3, Egr2 and Ier3) as well as novel (Enc1 and Bhlhe40) targets for GR-mediated repression that are largely unaffected by LPS treatment. Thus, this cluster is formally outside of group III.

#### IV) Genes downregulated by LPS

LPS-repressed genes were separated into 3 clusters based on the combined effect of Dex and LPS on gene expression. The LPS-mediated downregulation is either weakly potentiated (*cluster 12*) or antagonized (*cluster 11*) by Dex. The functions of the majority of these genes are poorly understood or unknown, however, 32% of genes in *cluster 11* and 20% in *cluster 12* encode uncharacterized C2H2 Zn-finger proteins implicated in transcription and chromosome maintenance.

Expression of genes in *cluster 10* is downregulated by Dex and LPS in an additive manner. At least one gene in this cluster (Angptl4) is a known GR target. Several genes encode regulators of immune cells activities including Rit1 and Cd300lb. *Cluster 9* contains Dex-induced genes that are weakly repressed by LPS including previously reported Ddit4, Arl4d and Sik1.

### Cluster validation by RT-qPCR

Two representative genes in each cluster were chosen for validation. Total RNA was isolated from treated (D, L, or L + D for 1 h) and control BMMФ and transcript levels for indicated genes (Figure 1) were determined by RT-qPCR using Act1 or Hprt as housekeeping control genes. As several LPS-induced, Dex-repressed genes coding for various cytokines that we found in clusters 3 (Tnf, Ccl2, 3 and 4), 4 (Il1a and b) and 5 (Lif, Niacr1, Mmp13) have been previously characterized by us and others [20–22], we focused on uncharacterized LPS/Dex targets. The expression patterns determined by RT-qPCR closely resembled those determined by RNA-seq, with the exception of several weakly expressed genes that were not detectably repressed by Dex following a 1-h treatment (*e.g*., Bcl2, Med21; data not shown).

### Glucocorticoid-regulated genes form a highly interconnected association network with distinct response-specific modules

To determine the prevalent functional patterns in groups of co-regulated genes, we performed gene enrichment analysis and visualization using GeneMANIA plugin [26] for Cytoscape 2.8 and Exploratory Gene Association Networks (EGAN) software [27].

**Figure 2:**
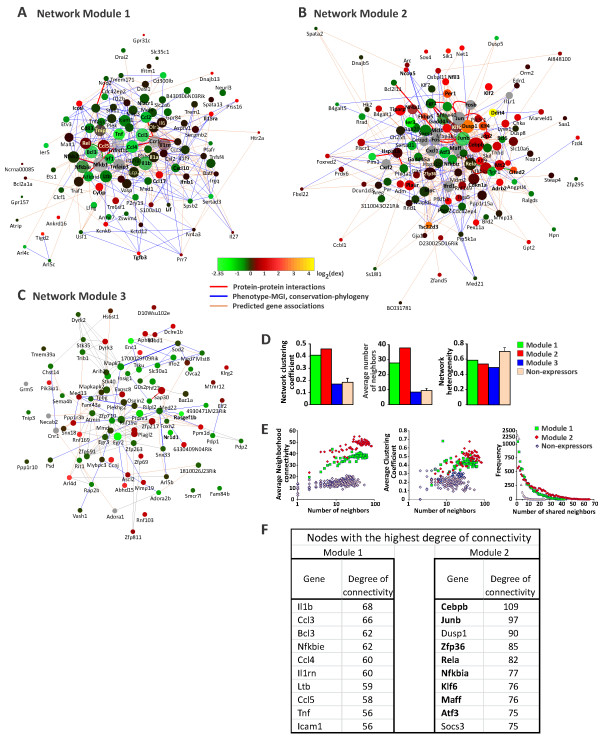
**Hormone-regulated genes in BMM** **Ф** **participate in highly interconnected networks with TF control nodes. (A)** Ranked list of Dex-responsive genes was used as an input for GeneMania network building algorithm. The resulting combined network was partitioned using the Newman-Girvan algorithm into three Modules enriched with **(A)** Dex-repressed, **(B)** Dex-induced and **(C)** LPS-repressed genes. To simplify the view, the edges representing co-expression, co-localization and shared protein domains were hidden. The size of the node is proportional to the node connectivity. The nodes are colored according to the magnitude of Dex effect on respective gene expression using MulticoloredNode Cytoscape plugin. The nodes predicted by GeneMania are colored gray. Both **(D)** network-wide and **(E)** node-specific network topological parameters in Modules 1 and 2 demonstrate high level of interconnectivity with a large number of shared nodes. **(F)** Nodes with the highest degree of connectivity in the Modules 1 and 2. The genes coding for TFs are shown in bold.

Using a list of genes that were up- (*clusters 1*, *6, 7* and *9*) and down- (*clusters 3*, *4*, *5*, *8* and *10*) regulated by Dex, we generated a consensus association network consisting of 333 nodes and 8296 edges. To discern underlying data structure in the Dex-regulated network, we decomposed this network using the Newman-Girvan community clustering algorithm [28], a divisive procedure that iteratively removes network edges with largest “edge betweenness”, recalculates this metric for a novel network and repeats the procedure until the network is split into several groups. The Newman-Girvan algorithm disregards edge weights and uses only connectivity to define communities. Community clustering partitioned Dex-regulated network into three unequal modules that were significantly enriched with Dex-repressed genes (Module 1, Figure 2A), Dex-induced genes (Module 2, χ^2^ = 18.33, p = 0.0001, Figure 2B) or LPS-repressed genes (Module 3, χ^2^ = 15.347, p = 0.00046, Figure 2C).Network topology analysis of Dex-responsive modules revealed significantly greater network densities and clustering coefficients in Modules 1 and 2 compared to networks generated from the same number of non-expressed genes extracted from the same BMMФ experiments (Figure 2D). Similarly, the average neighborhood connectivities and average clustering coefficients for Modules 1 and 2 were considerably greater than the values for non-expressors (Figure 2E), indicating that nodes in these modules form tight interconnected local groups. A broader shared neighbors distribution in Modules 1 and 2 indicates high prevalence of shared nodes, suggesting an enrichment for multiple input motifs. Interestingly, Module 2 (enriched with Dex-induced genes) was more structured than Module 1 (predominantly Dex-repressed genes) as evidenced by consistently higher average neighborhood connectivities and average clustering coefficients (Figure 2E). Although Module 3 has a non-random composition, with the exception of network heterogeneity, all other analyzed topological metrics were typical of networks composed of randomly selected non-expressed genes (Figure 2D). Therefore, we focused the rest of the analysis of Modules 1 and 2. Relatively high heterogeneity for both modules (0.506 and 0.581; Figure 2D) indicates the presence of network hubs - nodes with high degree of connectivity. Indeed, 10 most connected nodes in Modules 1 and 2 account for 36 and 32% of all edges, respectively (Figure 2F). Interestingly, eight out of 10 most connected nodes in Module 2 are sequence-specific DNA-binding TFs (bold in Figure 2F).

### Glucocorticoid response-specific modules are functionally distinct

**Figure 3:**
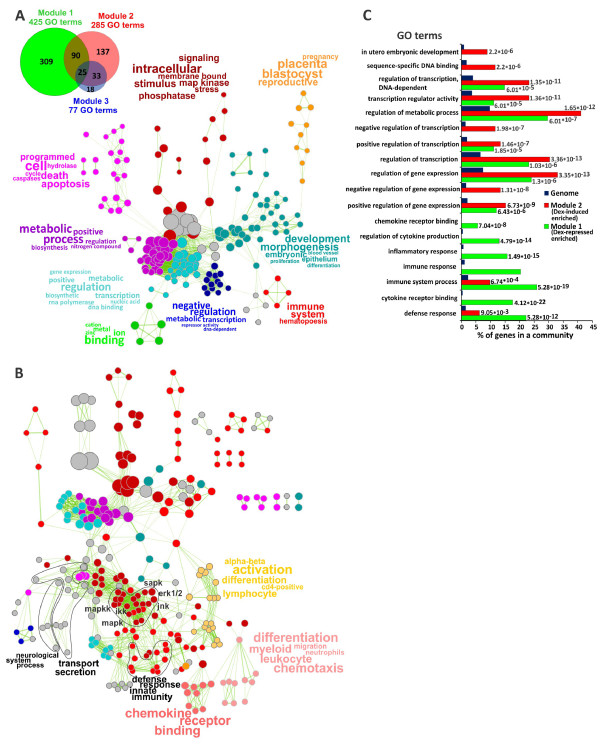
**Distinct functional GO categories are enriched in Dex-regulated network Modules in BMM** **Ф** **.** GO categories enriched among Dex-regulated genes in Modules 2 **(A)** and 1 **(B)** (p < 10^-3^, hypergeometric test) were used to create GO terms similarity networks where the nodes represent GO gene sets and the edge length between two nodes is proportional to the fraction of shared genes in categories. Individual clusters were manually repositioned to simplify network layout and colored as indicated; the broad top GO terms are colored grey. The word tags were generated with WordTag Cytoscape plugin [29] to represent frequency-based semantic summary of words in GO category titles found in individual clusters and the size of the nodes is proportional to the number of genes in the respective GO gene set. **(C)** GO enrichment analysis of individual GO categories in Modules 1 and 2 relative to the mouse genome. The FDR-corrected p-values (hypergeometric test) are shown for each bar.

Using gene ontology (GO) analysis to determine enriched gene categories in subsets of functionally related genes, we identified 425 enriched GO categories for Module 1, 285 for Module 2 (FDR corrected p < 10^-3^) and 77 for Module 3 (FDR corrected p < 10^-2^). Only 115 GO categories overlapped between Modules 1 and 2 (Figure 3A, 3C). To facilitate visualization and interpretation of these results and compare enriched functional categories among groups of Dex-regulated genes, we generated GO terms similarity networks using Gene Set Enrichment Mapping Cytoscape plug-in. Multiple GO categories related to regulation of metabolic processes, embryonic and post-embryonic development and regulation of apoptosis and signaling are enriched in Module 2 (Figure 3A) that contains a large number of Dex-upregulated genes. Notably, 32/285 GO categories enriched in Module 2 were related to regulation of gene expression, regulation of transcription, sequence-specific DNA binding transcription factors. For example, negative regulation of gene expression (GO:10629), negative regulation of transcription (GO:16481), negative regulation of transcription - DNA-dependent (GO:45892), sequence-specific DNA binding (GO:43564), negative regulation of transcription from RNA polymerase II promoter (GO:122) and transcription regulator activity (GO: 30528) were all enriched in Module 2, but not Module 1 (Figure 3C). Overrepresentation of genes coding for regulators of gene expression in the early Dex-responsive transcriptome suggests that GR initiates a transcriptional program that relies on the step-wise activation of multiple TFs. Only a few categories related to immune/inflammatory responses have been found in Module 2 (Figure 3A).

Conversely, the majority of enriched GO categories in Module 1, which contains predominantly Dex-repressed genes, are related to immune and inflammatory responses, signaling and regulation of signal transduction and metabolic regulation including immune response (GO:6955), immune system process (GO:2376), inflammatory response (GO:6954) and regulation of cytokine production (GO:1817) (Figure 3B, 3C). The fraction of gene expression-related GO categories in this module is significantly smaller (22/425, χ^2^ = 8.05, df = 1, p = 0.00455) than in Module 2.

### Dex-responsive transcription regulators

**Figure 4:**
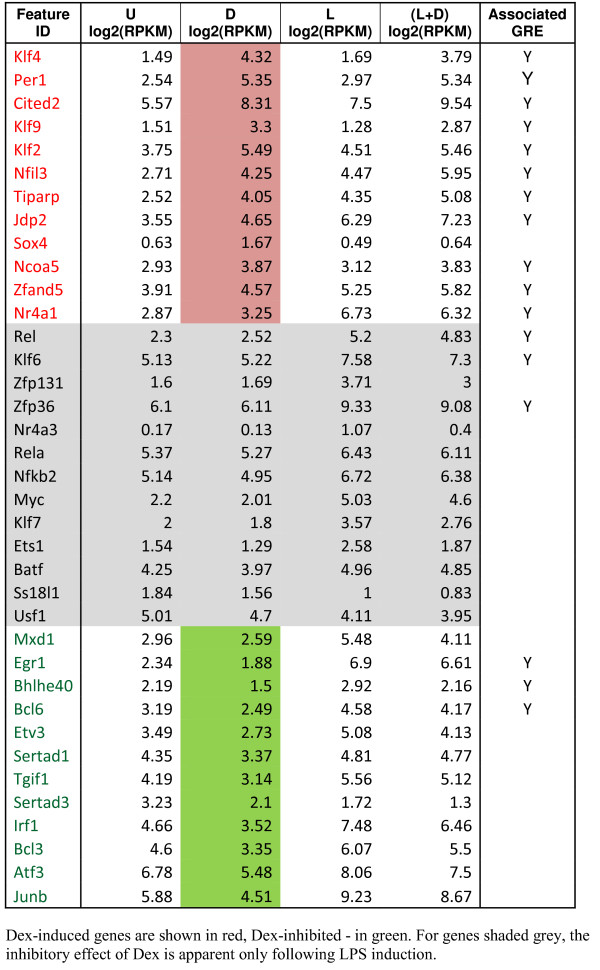
**Dex-responsive regulators of gene expression.**

**Figure 5:**
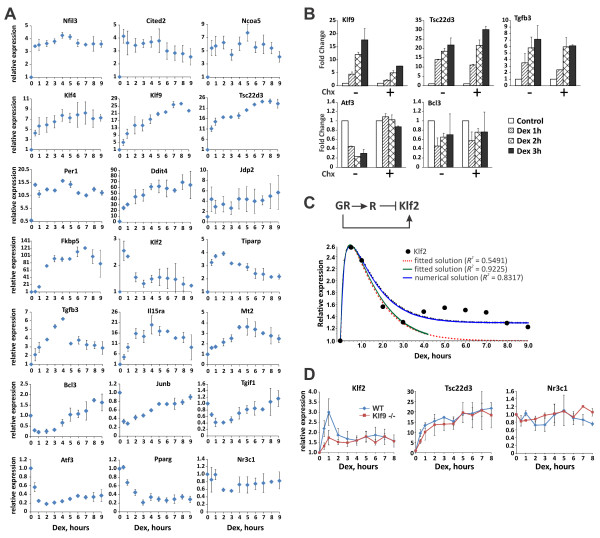
**The dynamics of hormone-responsive gene expression is consistent with FFL network motifs. (A)** BMMФ were treated with 100 nM Dex for indicated time and the expression levels of Dex-responsive genes were determined by RT-qPCR. **(B)** BMMФ were treated with Dex either alone or in the presence of the protein synthesis inhibitor Chx for 1–3 h; the expression of indicated genes were determined by RT-qPCR and expressed relative to transcript levels in the absence of Dex (‘Control’), with or without Chx (=1). **(C)** The dynamics of Klf2 expression is consistent with the I1-FFL with a strong repressor (R) as an intermediate regulator. Klf2 expression data (black circles) collected over 9 h was subjected to a global least square analysis (red line) using the equation (1) (Additional file 1). The quality of the fit as determined by calculating coefficient of determination R^2^ (Additional file 1) improved considerably when the expression data were limited to initial 4 h (green line). The numerical solution of the equation (3), which allows for variation in degradation rates of both “R” and Klf2, yields a better fit (blue line) to the Klf2 expression data. The R^2^ for curve fitting analyses are shown in the legend. **(D)** Glucocorticoid induction of Klf2 in BMMФ derived from Klf9-KO mice (red squares) loses peak-like kinetics, characteristic of I1-FFL controlled genes (blue diamonds). WT and Klf9-KO BMMФ were cultured in the presence of Dex for indicated time and the expression of Klf2, Tsc22d3 and Nr3c1 (GR) was assessed by RT-qPCR as in A. Basal levels of each transcript were set to 1 for untreated BMMФ of each genotype. Error bars are standard errors of the mean.

We identified 37 Dex-responsive genes whose products are involved in the regulation of gene expression (Figure 4). 12 of these genes including TFs Klf2, 4 and 9, Per1, Jdp2, Cited2, Nfil3 and Tiparp are upregulated by Dex; 12 others including TFs Junb, Atf3, Tgif1, Irf1 and Bcl3 are downregulated; for 13 regulatory proteins (*e.g*., Zfp131, Zfp36, Nr4a3, Rela, Nfkb2, Klf7 and Ets1), the inhibitory effect of Dex is apparent only in LPS-induced MФ. For all Dex-induced and a subset of Dex-repressed TFs, we have independently confirmed RNA-seq data by RT-qPCR (Additional file 3: Figure S1; also see Figure 1 for Cited2, Irf1, Etv3 and Atf3).To uncover potential functional interactions between Dex-regulated TFs, we treated BMMФ with Dex for up to 9 h and determined expression levels of a subset of Dex-regulated genes by RT-qPCR. We observed a striking difference in expression patterns over time. Nfil3, Cited2, Jdp2 and Per1 (Figure 5A) are characterized by an accelerated burst phase, with the mRNA level reaching maximum within 30–60 min and then remaining constant (Nfil3 and Cited2) or slowly declining over time (Per1 and Ncoa5). Klf4, Klf9, Tsc22d3 and Ddit4 are strongly induced within the first 2 h, and their RNA levels continue to increase for the next 6 h (Figure 5A). The expression profile of Fkbp5 exhibits an initial delay, followed by a robust and sustained induction (Figure 5A). Conversely, Klf2 and Tiparp displayed pulse-like rapid upregulation within 1–3 h followed by a decline in transcript level, which in the case of Klf2 reaches baseline (Figure 5A); a similar biphasic pattern of expression was observed for Tgfb3, Il15ra and Mt2 (Figure 5A). Interestingly, Bcl3, Junb and Tgif1 responded with rapid pulse-like downregulation followed by a slow return to basal expression level, whereas Atf3 was rapidly downregulated within the first hour and remained repressed throughout the time course (Figure 5A). Unexpectedly, the Pparg expression was only modestly induced by Dex at the early time points, then decreased dramatically by 3 h and remained low for up to 9 h (Figure 5A).

The dynamics of expression for several Dex-regulated TFs suggests that they are under combinatorial controls that involve GR and additional GR targets which either cooperate with or antagonize GR actions. As such a model implies transcription/protein production of these putative GR targets, we first examined the expression of Dex-regulated genes in the presence of a protein synthesis inhibitor cycloheximide (Chx). Treatment with Chx up for to 3 h had no dramatic effects on Dex-mediated regulation of Klf9, Tcs22d3, Tgfb3 and Bcl3 (Figure 5B), suggesting a direct regulation by GR that does not rely on synthesis of additional proteins. Conversely, the expression of Atf3 became refractory to Dex in the presence of Chx (Figure 5B) suggesting that additional proteins induced by Dex rather than GR itself are likely to directly regulate this gene. For several Dex-responsive genes (Klf2, Klf4, Nfil3, Tiparp, Tgif1), however, treatment with Chx dramatically upregulated their basal expression, complicating the assessment of the relative contribution of direct vs. indirect effects of GR to their regulation (Additional file 3: Figure S2) and necessitating an alternative approach.

### Temporal dynamics of hormone-regulated gene expression is consistent with feed forward logic

The dynamics of the transcriptional response of several genes to Dex imply the existence of some feedback mechanism that limits activation by GR yet, at the same time, is GR-dependent. Because Chx elicits many off-target effects and does not enable us to discriminate between the secondary targets of GR and those jointly controlled by GR and a GR-regulated TF, we performed dynamic modeling of expression data in an attempt to discern specific regulatory patterns. Several mechanisms including positive and negative autoregulation, positive and negative feedback and feed forward loops (FFL) could account for deviations from a simple model with a single TF regulating gene expression via a single DNA binding site [12]. The kinetics of GR expression following Dex stimulation is not consistent with auto-regulatory models (Figure 5A). Depending on the overall regulatory outputs and activities of individual FFL components, two types of FFL have been recognized – coherent (C-FFL) and incoherent (I-FFL). In type 1 I-FFL (I1-FFL), the activating master TF and a repressing intermediate regulator have opposite effect on a jointly regulated gene. Dynamic modeling and experimental studies of I1-FFL dynamics have demonstrated several properties of this network motif, including its ability to produce sharp pulse-like activation of a jointly regulated gene with a fast relaxation time [11, 13]. Several GR-regulated genes, including Klf2, Tiparp, Tgfb3, Mt2 exhibit pulse-like kinetics at constant Dex exposure (Figure 5A). Near-baseline relaxation of Klf2 expression suggests that this gene is under control of both GR and a strong Dex-induced repressor. Because GR is largely inactive in the absence of ligand, glucocorticoids act as a low-latency on-off switch eliminating the need to correct for a baseline activity of GR. The dynamics of Klf2 and repressor (R) expression is described by the ordinary differential equations (2) and (3) (see Additional file 1) [11, 13].

Assuming equal degradation rates of Klf2 and R, these equations can be solved analytically (equation (1) and [13]) and used to fit the expression data for Klf2. When limited to the early data points (up to 4 h), the expression data fit very well to the predicted expression pattern (Figure 5C, *R*^*2*^ = 0.9225), however, at the later time, when the contribution of degradation rates becomes significant, the quality of fit decreases (*R*^*2*^ = 0.5491). Using parameter estimates derived from equation (1) fitting, we solved equations (2) and (3) numerically. Figure 5C shows a good concordance between the experimental data for Klf2 expression as determined by RT-qPCR and the numerical solution (*R*^*2*^ = 0.8317) that describes the dynamics of the I1-FFL. This result strongly suggests that Klf2 and, potentially, several other GR targets that exhibit similar expression dynamics (Tiparp, Tgfb3 and Mt2) are jointly regulated by GR and a GR-induced repressor via the I1-FFL network motifs.

Numerical solutions of equations 2 and 3 also provide a theoretical prediction of an intermediate repressor dynamics in the GR-R-Klf2 I-FFL. Interestingly, among several known transcription repressors activated by GR, the expression kinetics of Klf9 fits the best to the predicted model (Additional file 3: Figure S3). The dynamics of the GR-R-KLF2 FFL can be tested by perturbing the concentration of a hypothetical intermediate repressor which should uncouple the FFL thus shifting peak-like FFL-mediated kinetics to simple monotonous kinetics eventually converging to a steady-state level. To test the role of Klf9 as a potential GR-activated repressor of Klf2 transcription, we derived M from Klf9-KO mice [30] kindly provided by Dr. Simmen and treated them with 100 nM Dex as above. Interestingly, in Klf9 null BMMФ the peak-like Klf2 induction profile “degenerated” to monotonous activation kinetics that plateaued at a steady state level by 3 h (Figure 5D), replicating the profiles of previously reported uncoupled experimental I-FFLs [11, 31], consistent with the proposed role of KLF9 as an intermediate repressor in the GR-KLF9-KLF2 I-FFL. At the same time, deletion of Klf9 did not affect the expression dynamics of either GR itself or the GR target gene Tsc22d3 with a simple monotonous activation profile (Figure 5D).

### GR is recruited to binding sites associated with Dex-regulated genes

**Figure 6:**
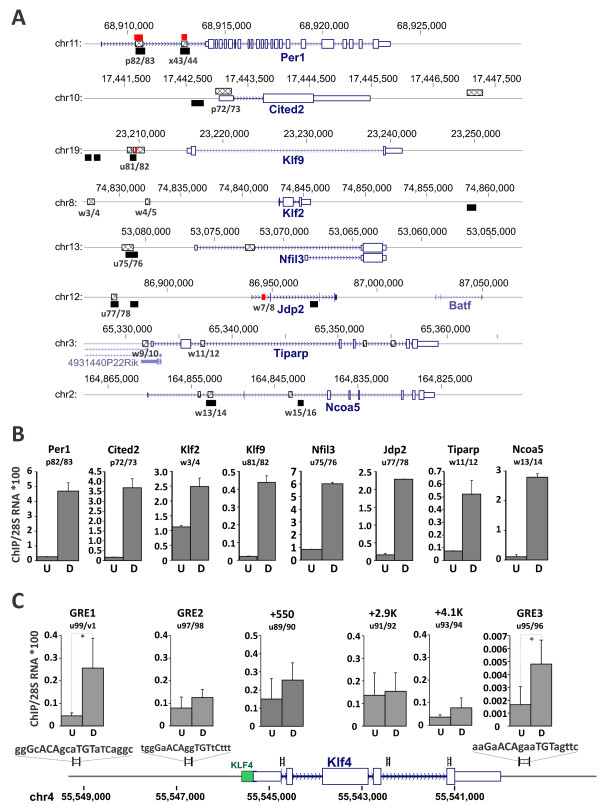
**GR binds genomic sites associated with genes encoding Dex-induced TFs. (A)** ChIP-seq data from BMMФ (black; see also Additional file 3: Figure S4), adipocytes (crosshatched) and C2C12 cells (red) reveal multiple Dex-dependent GR binding peaks associated with Dex-induced genes encoding TFs. The numbers under the putative GR binding sites indicate primer pairs used in ChIP-qPCR experiments in BMMФ (Additional file 2: Table S3). **(B)** ChIP-qPCR-assessed GR occupancy at putative GR binding sites indicated in (A) prior (U) and upon (D) a 40-min stimulation with 100 nM Dex. **(C)** Scanning ChIP of the Klf4 genomic region reveals a putative GR binding site. Primer positions and amplified products are shown as black rectangles over the gene schematics. Putative sites harboring sequences similar to GR binding sites are labeled as GRE1,2,3 with the binding sequence shown on top. The capital letters indicate nucleotides fitting a consensus GRE. A green rectangle overlapping the Klf4 TSS indicates the position of a KLF4 binding site identified in [36]. Data for each site represent average of 2 or more independent experiments; error bars are standard deviation.

**Figure 7:**
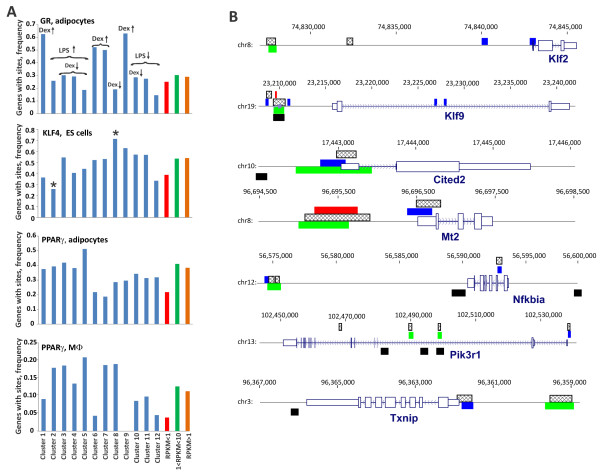
**The distribution of GR, KLF and PPAR**γ **binding sites at Dex/LPS-responsive genes in BMMФ. (A)** For each expression *cluster* (Figure 1), the frequencies of genes with binding sites for at least one of the TF (GR [32], KLF [36] or PPARγ [38, 39]) within the region encompassing the gene and 15 Kb of flanking sequences on each side (blue) was compared to those in random group of non-expressors (RPKM < 1, red), low expressors (1 < RPKM < 10, green) and all expressing genes (RPKM > 1, orange) in BMMФ. **(B)** A select group of Dex-responsive genes contains clusters of GR (black - macrophages (this study), crosshatched - adipocytes [32], red - C2C12 cells [33]), KLF4 (blue; [36]) and PPARγ (green; [38]) binding sites.

The FFL gene regulatory circuitry predicts that the master TF binds DNA to regulate transcription of both FFL nodes. Using several published mouse ChIP-seq datasets of acute GR recruitment [32, 33] we interrogated Dex-induced genes for the presence of GR binding sites within the gene and 15 Kb upstream and downstream of the gene. With the exception of Sox4 and Klf4, all genes encoding Dex-induced TFs contained at least one peak within the analyzed intervals (Figure 6A, Figure 4). To compare these peaks to those in MФ, we analyzed GR recruitment by ChIP-seq using untreated MФ as a control and identified 16,657 peaks induced by a 40-min Dex exposure at 2% FDR. Selective comparison of binding site distributions revealed a high level of concordance between Dex-induced peaks in MФ and those previously described in adipocytes [32] (Figures 6A, 7B and Additional file 3: Figure S4) and a partial overlap with a GR cistrome in MФ polarized with high dose long-term glucocorticoid exposure [34]. By ChIP-qPCR, we detected GR recruitment as early as 40 min post Dex treatment at multiple putative GR binding sites, including those at Per1, Cited2, Klf2, Klf9, Nfil3, Jdp2, Tiparp and Ncoa5 (Figure 6B). These observations correlate well with the expression data (Figures 1 and 5A). Although Klf4 was strongly induced by Dex, no glucocorticoid response elements (GREs) near the gene has been previously reported. We performed a scanning ChIP with evenly spaced primers within the Klf4 gene and several primers flanking potential GR binding sites (Figure 6C). Two of the putative GREs located at -3830 bp (gGcACAgcaTGTaTC) and +5896 bp (aGaACAgaaTGTagttc) relative to the Klf4 transcription start site recruited GR following a 40-min treatment with Dex (Figure 6C), consistent with the notion that, similar to genes shown in Figure 6B, GR is likely to regulate Klf4 directly.

We then used a set of Dex/LPS-regulated genes to analyze the distribution and enrichment of binding sites for TFs that were present in Chip-Enrichment Analysis (ChEA) database [35]. The ChEA database contains curated published genome-wide datasets of TF binding sites in human, mouse and rat. After filtering out TFs that were not expressed in MФ (RPKM < 1), we noted that binding sites for several Dex-responsive TFs, such as KLF2, KLF4, ATF3, EGR1, CEBPβ and IRF1 are enriched among Dex/LPS-regulated genes. Interestingly, binding sites for PPARγ, whose expression was inhibited upon prolonged Dex treatment, were found near the majority (22/37) of Dex-responsive gene expression regulators (Figure 4) and highly enriched among Dex/LPS-regulated genes in general.

We then determined the frequency of genes associated with binding sites for several TFs identified by ChEA and induced by Dex in individual clusters of Dex/LPS-regulated genes (Figure 1). We defined a binding site as ‘gene-associated’ if its genomic intervals overlapped with genomic intervals encompassing all mouse genes annotated in mm9 +/- 15 Kb by at least one nucleotide. In good correlation with the RNA-seq data, acute GR recruitment peaks previously identified by ChIP-seq in Dex-treated adipocytes [32] were enriched in Dex-induced *clusters 1*, *6*, *7* and *9* (Figure 7A, arrow up). Among Dex-regulated TFs, mouse genome-wide binding datasets are currently available for KLF4 (ChIP-seq), KLF2 (chip-on-chip), PPARγ (ChIP-seq) and NFIL3 (chip-on-chip) [36–40]. Although KLF4 sites are enriched in the entire Dex/LPS dataset compared to non-expressing genes, only in *cluster 8* (Dex-repressed genes) the enrichment level attains significance (Figure 7A). Conversely, KLF4 binding sites are underrepresented in the LPS-induced *cluster 2*. Although the KLF2 chip-on-chip dataset available was relatively small, several KLF2 targets were regulated by Dex and contained GR binding sites, including Klf2 itself, Klf4, Klf9 and Tgfb3 [37].

Two mouse PPARγ datasets, one from differentiated adipocytes and one from resting MФ are currently available [38, 39]. Consistent with the previously reported role of PPARγ in repression of inflammatory genes, PPARγ binding sites are overrepresented in several LPS-induced clusters (*clusters 2*, *3* and *5*; Figure 7A) in both datasets. In addition, in the MФ dataset, PPARγ binding sites were enriched in Dex-induced and -repressed *clusters*, *7* and *8*, respectively (Figure 7A).

The only available genome-wide dataset of Nfil3 binding was acquired in a neuronal cell line [40]. Among Nfil3-occupied genes identified in that study, only four genes overlap with Dex/LPS-regulated dataset reported here; however, one of them, Tsc22d3 (GILZ), is a well-characterized GR target.To identify genes that might be under combinatorial control by GR and another Dex-responsive TF, we searched for loci that contained GR, KLF and/or PPARγ binding sites located close to each other within a gene +/- 15 Kb. Intriguingly, several GR targets including Klf2, Klf9, Cited2 and Mt2 contained tight clusters of binding sites for GR, KLF(4) and PPARγ (Figure 7B) suggesting that these TFs may interact functionally or physically at a subset of GR-regulated genes.

### LPS downregulates a unique class of genes encoding the C2H2-KRAB gene expression regulators

**Figure 8:**
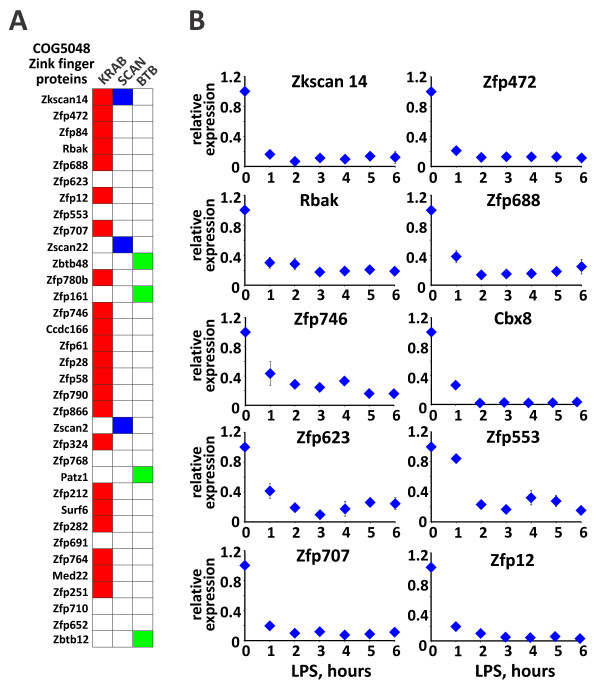
**Many LPS-downregulated genes encode Zn-finger proteins containing KRAB domains. (A)** Domain overrepresentation analysis among LPS-suppressed genes (*Clusters 10*, *11* and *12*) revealed a large number of Zn-finger proteins (COG 5048) that, in addition, contain KRAB, SCAN and BTB domains. The list of LPS-downregulated Zn-finger proteins is shown with KRAB, SCAN and BTB domains indicated as red, blue and green boxes, respectively. **(B)** The kinetics of LPS-mediated suppression of select genes encoding Zn-finger KRAB domain-containing proteins. BMMФ were treated with LPS for indicated time and the expression of indicated genes was assessed by RT-qPCR.

Even a brief LPS treatment results in a marked downregulation of a large number of genes confined to *clusters 10–12*. GO overrepresentation analysis of LPS-repressed genes revealed that many of them participate in the regulation of nucleic acid metabolism, gene expression and transcription however, detailed information on specific functions of many of these proteins is lacking. Interestingly, 33 proteins in these clusters contained tandem zinc-finger motifs (COG:5048, p = 3.49*10^-29^, Figure 8A). We confirmed that the expression of 10 of these Zn-finger proteins is rapidly downregulated by LPS in MФ, and remains suppressed for up to 6 h of treatment (Figure 8B). Further analysis of domain architecture revealed that in the majority of these proteins, tandem Zn-fingers co-occur with domains such as Kruppel-Associated Box (KRAB, Pfam01352, p = 1.029*10^-19^), BTB (Pfam00651, p = 5.68*10^-5^) and SCAN (Pfam 02023, p = 2.1*10^-4^, Figure 8A). KRAB is a *Tetrapoda*-specific domain that defines one of the largest sub-families of Zn-finger proteins [41] which are involved in nucleic acid binding and regulation of gene expression. Although the specific functions of the majority of KRAB proteins with respect to innate immunity are not well studied, in a few characterized cases KRAB proteins have been associated with transcriptional repression, establishing reversible patterns of histone and DNA methylation and reversible heterochromatization [42–44].

## Discussion

### Glucocorticoids- and LPS-regulated gene expression programs

GR is a ubiquitous ligand-dependent TF capable of eliciting highly divergent transcription programs with up to a third of protein-coding genes differentially expressed following a 24-h glucocorticoid treatment [7]. Establishing the hierarchy of regulatory events upon prolonged hormonal exposure in individual cell types is challenging, which complicates both accurate mechanistic predictions and clinical decisions. Multiple GR ligands have been designed in an attempt to create a highly specific compound that selectively regulates desired subsets of genes. Mechanistic analyses of these ligands usually focus on a specific group of disease-relevant genes and often involve long-term treatments, which obscure primary and transient responses to GR activation by a plethora of secondary pathways. In the context of inflammation, both immediate and delayed regulatory events are clinically relevant as they reflect typical glucocorticoid treatment modalities. We reasoned that by analyzing early transcriptomes elicited by the inflammatory and glucocorticoid exposure in MФ, a clinically relevant cell type, we will be able to isolate a key set of immediate GR targets responsible for the delayed gene expression patterns. Our results indicate that early glucocorticoid- and LPS-dependent changes establish a highly organized program of gene expression with distinct groups of genes following cooperative and antagonistic regulation. As expected from previous work [22, 34, 45] a large group of LPS-induced genes that included among others inflammatory cytokines, was rapidly downregulated by glucocorticoids. Another group encompassing glucocorticoid-induced genes, some of which encode TFs (Cited2, Nfil3, Jdp2) or signaling proteins (Dusp1, Tsc22d3), are involved in curbing inflammatory signaling [46]. We identified several previously unreported glucocorticoid-induced genes whose products are involved in signaling (Ccl17, Il15ra and Fzd4), regulation of transcription (Klf2, Jdp2, Ncoa5 and Tiparp) and mRNA stability (Zfand5). Several of these genes add to the arsenal of anti-inflammatory mediators regulated by GR. For example, KLF2 interferes with AP1- and NFκB-mediated transcription of Tnf and several chemokines including Ccl2, 3 and 4 [47]. Furthermore, Klf2 haploinsufficiency in mice results in an exaggerated inflammatory response and more severe disease in arthritis models [48]. CCL17, another previously unreported glucocorticoid-induced chemokine, is a marker and promoter of the polarization of ‘alternatively activated’ M2 MФ, which are considered anti-inflammatory and mediate tissue repair and wound healing [49, 50]. In addition to repressing cytokine gene transcription, glucocorticoids downregulate expression of several TFs including Atf3, Junb, Irf1, Bcl3, Tgif1, some (*e.g.*, Rela, Nfkb2, Myc, Ets1) in the context of LPS induction, and, unexpectedly, Pparg An enrichment in positive regulators of inflammation and cell proliferation among Dex-downregulated TFs is consistent with the anti-inflammatory and anti-proliferative effects of glucocorticoids. The role of GR in repression of the Pparg gene in MФ has not been previously reported, the effect might be indirect and mediated by a well-established GR target GILZ [51], which may also account for the delay (Figure 5A). Finally, we described a previously overlooked group of LPS-downregulated genes encoding proteins with the C2H2 Zinc-fingers adjacent to the KRAB domain. Despite being one of the largest TF family, KRAB proteins remain poorly characterized. Among those whose functions were described, several are involved in transcriptional regulation, RNA and DNA binding and splicing [42–44, 52]. KRAB domains interact with a scaffolding co-repressor TRIM28 (KAP1, TIF1β) which in turn binds the heterochromatin protein 1, chromatin remodeler MI2A and H3K9-specific methyltransferase [53]. Indeed, some KRAB proteins reportedly repress transcription by heterochromatin spreading [52]. Interestingly, several KRAB proteins have been linked to NR actions [54, 55]. The role of KRAB proteins in inflammation is essentially unknown; however, genomic studies indicate that inflammatory signaling increases accessibility of large sections of the genome [56]. It is tempting to speculate that a broad downregulation of proteins involved in heterochromatin maintenance and spreading serves to increase DNA accessibility and inflammatory gene transcription.

### The dynamic response to GR activation is consistent with feed forward logic

Functional relationships between GR and its targets are often classified as “direct”, that involve GR recruitment to genomic binding sites associated with regulated genes, and “indirect”, whereby primary GR-regulated factors, rather than GR itself, are responsible for activation of the downstream targets. Thus, the activation of these secondary targets is often described as sequential or delayed. Such a model, however, cannot explain many instances of non-monotonous expression dynamics (see Figure 5) and non-linear response to varying hormone concentration of many GRE-driven genes [57]. The large number of shared neighbors, overrepresentation of TFs and their high interconnectivity in GR regulatory networks (Figure 2) are consistent with more intricate regulatory modalities such as FFL. Variations in kinetic parameters for participating TFs, target gene structure and activation/repression thresholds often lead to paradoxical responses to stimulation of the master TF with profound functional implications. C-FFLs serve as delayed response organizers that detect the duration/strength of a signal that activates the initiating TF [11, 12]. Interestingly, the dynamics of Fkbp5 induction by Dex, characterized by a substantial post-exposure delay followed by a robust expression (Figure 5A), is reminiscent of the C-FFL in which the jointly regulated gene is activated by both the master and intermediate TFs [12]. Although additional experiments are required to establish the precise mode of Fkbp5 regulation, this gene is a known direct GR target that recruits GR to several GREs (Additional file 3: Figure S5).

Incoherent loops are responsible for negative and positive pulse generation, accelerated response and fold change sensing [11, 13, 14]. Here, we observed that several GR target genes exhibit both positive (Klf2, Tiparp, Tgfb3 and Mt2) and negative (Tgif1, Junb and Bcl3) pulse-like dynamics consistent with the I-FFL. In keeping with the role of a potential master regulator, GR binds to the GREs in regulatory regions of many of these genes (Figure 6). Furthermore, using a system of ordinary differential equations which describe FFLs in the “fold sensors” model [13], we showed that Klf2 expression is consistent with that of a gene under joint control of GR and a strong GR-activated repressor (Figure 5C). Several GR-activated genes are either known transcription repressors (*e.g*., Klf4, 9, Nfil3, Per1 and Jdp2) or may downregulate gene expression by destabilizing RNA transcripts (Zfand5, [58]). Curiously, the expression dynamics of Klf9 fits closely with the computational prediction of an intermediate repressor in the GR-R-Klf2 I-FFL (Additional file 3: Figure S3). GR is recruited to the Klf9 and Klf2 GREs as early as 40 min of Dex treatment. Both Klf9 and Klf2 regulatory regions also contain functional GAGGCGTGG KLF sites ([36], Figure 7B) which can be occupied by various TFs of the KLF family [59] including KLF9. Finally, in KLF9-KO macrophages, the induction profile of Klf2 loses the early peak followed by a decrease and acquires monotonous kinetics (Figure 5D) strongly suggesting a collapse of the I1-FFL to simple GR-dependent activation. Interestingly, KLF binding sites are overrepresented in glucocorticoid-regulated genes and are located near GREs in several *bona fide* GR target genes suggesting that these factors may co-regulate a number of GR targets.

### KLF proteins in inflammation

Both KLF2 and KLF4 have been implicated in myeloid cell biology. KLF2 inhibits monocyte activation by inhibiting NFκB activity, which correlates with decreased expression of multiple cytokines and HIF1α, a TF that regulates myeloid cell response to bacterial infection and reactive oxygen species [60]. Consistent with the anti-inflammatory role of KLF2, mice hemizygous for Klf2 have elevated levels of inflammatory mediators, such as CCL2 and PTGS2 (COX-2). By extension, in KLF2-deficient mice, the manifestations of both Me-BSA- and IL1β-experimentally induced inflammatory arthritis are more severe [48].

KLF4 is involved in inflammatory monocyte differentiation [61, 62] and in MФ polarization toward the M2 anti-inflammatory phenotype [63]. KLF9 can act as either a transcriptional activator or a repressor [64, 65], however its role, if any, in inflammation has not been described. We showed here that GR regulates Klf genes with distinct temporal dynamics and proposed that KLF9 may act as a GR-induced Klf2 repressor. Thus, it is tempting to speculate that GR anti-inflammatory activities rely in part on the activation of Klf genes whose products regulate transcription of additional targets in concert with GR. Indeed, glucocorticoids and KLF4 regulate partially overlapping set of genes during epidermal barrier establishment in embryogenesis [66]. The proximity of the GREs and KLF binding sites in the genome suggests an intriguing possibility that GR and KLFs interact functionally or physically. Curiously, a functional interaction with the I-FFL logic has been reported for GR and another member of KLF family, KLF15 [67]. Although KLF15 is not expressed in MФ, our studies strongly suggest extensive crosstalk between GR and other KLF family members in the innate immune cells.

## Conclusions

Anti-inflammatory activities of glucocorticoids involve downregulation of inflammatory mediators and activation of various anti-inflammatory genes. The early glucocorticoid-driven transcriptome in MФ contains an unusually large number of genes coding for transcriptional regulators. Temporal dynamics of hormone-regulated gene expression is consistent with feed forward logic suggesting that GR and GR-induced TFs jointly regulate GR target genes. In particular, our data suggest that GR is rapidly recruited to and activates genes encoding several members of the KLF family of TFs with profound anti-inflammatory activities, such as Klf2 and Klf4. Furthermore, GR appears to regulate Klf2 expression via the GR-Klf9-Flf2 I1-FFL. We propose that by acting as a hub for highly branched regulatory networks and activating genes encoding TFs to propagate the initial signal, GR coordinates anti-inflammatory responses.

## Methods

### Mice and macrophage cultures

C57BL/6 mice (NCI, Charles River Laboratories) were maintained in the Hospital for Special Surgery Animal Facility in full compliance with institutional guidelines approved by the HSS Animal Care and Use Committee. Klf9-KO mice [30] were generously provided by Dr. R. Simmen (U. of Arkansas). BMMФ were prepared from 8–12 weeks old mice as in [23]. For RNA-seq, BMMФ from two independent mice were treated with vehicle, Dex (100 nM), LPS (10 ng/ml) or LPS + Dex for 1 h. For qPCR and ChIP analyses, BMMФ were treated as above for time indicated in Figure Legends.

### RNA isolation, RT-qPCR and RNA-seq

Total RNA was isolated using the RNeasy kit (Qiagen). 0.25 μg of total RNA was used for random primed cDNA synthesis which was performed with M-MuLV reverse transcriptase (NEB) according to the manufacturer’s recommendations. Quantitative PCR (qPCR) was performed using Maxima Sybr Green/ROX/ 2x master mix (Fermentas) on StepOne Plus real time PCR system (ABI) and analyzed using δδ*Ct* method as described previously [22] with Hprt or Act1 as a normalization control. Primer pairs are listed in Additional file 2: Table S3. RNA-seq is described in (Additional file 1).

### ChIP-qPCR and ChIP-seq

BMMФ were incubated -/+100 nM Dex for 40 min and ChIPs were performed as in [23] using N499 [22] and sc1004 (Santa Cruz Biotechnology) anti-GR antibodies, or normal rabbit IgG as a background control. The data for each recruitment site was normalized to non-specific signals at the unrelated 28S ribosomal gene. Primer pairs are listed in (Additional file 2: Table S3). ChIP-seq is detailed in (Additional file 1).

### Gene association network construction, analysis and expression data modeling

GeneMANIA algorithm was used to build gene association networks. Starting from a gene list of interest (*e.g*., combined list of Dex up- and downregulated genes), geneMANIA algorithm creates a consensus network and predicts gene functions based on integration of multiple prebuilt gene association networks. The detailed description of Gene association network construction, analysis and expression data modeling is in the Supplemental Information section.

## Electronic supplementary material

Additional file 1: **Supplemental methods and references.** (PDF 164 KB)

Additional file 2: Table S1: Contains statistical summary of combined RNA-seq experiments. **Table S2.** contains a detailed summary of genes regulated in BMMΦ upon a 1-h treatment with Dex (D), LPS (L) and LPS + Dex (L + D) compared to untreated control (U). **Table S3.** contains RT-qPCR and ChIP primers used in this study. (PDF 265 KB)

Additional file 3: Figure S1: Is RT-qPCR confirmation of RNA-seq expression data for Dex-responsive genes encoding TFs. **Figure S2.** shows the effect of Chx on the basal and Dex-regulated levels of GR-responsive transcripts. **Figure S3.** shows the dynamics of Klf9 induction by Dex compared to the predicted expression of a putative Klf2 repressor in the GR-induced I1-FFL. **Figure S4.** shows ChIP-seq data for acute Dex-induced GR recruitment to genomic binding sites in mouse macrophages. **Figure S5.** demonstrates that GR binds to multiple sites within the Fkbp5 gene in response to Dex. (PDF 1 MB)

## Abbreviations

Dex: Dexamethasone
GR: Glucocorticoid receptor
GRE: Glucocorticoid response element
BMMФ: Bone-marrow derived macrophages
LPS: Lipopolysaccharide
NR: Nuclear receptor
TF: Transcription factor
FFL: Feed forward loop
RT-qPCR: Reverse transcription coupled with quantitate polymerase chain reaction
ChIP: Chromatin immunoprecipitation.
