# Supplementary material for: Glucocorticoid receptor coordinates transcription factor-dominated regulatory network in macrophages
Source: BMC Genomics. 2014 Aug 6;15(1):656. doi: 10.1186/1471-2164-15-656 (PMC4133603; doi:10.1186/1471-2164-15-656)
Supplement: Supplementary file 1 — Additional file 1: Supplemental methods and references. (PDF 164 KB) [file 12864_2014_6351_MOESM1_ESM.pdf]

Supplemental Methods

RNA-seq

5 μg of total RNA was polyA-enriched, fragmented and converted into a library of Illumina-compatible sequencing templates with Illumina mRNA-seq sample preparation kit according to the manufacturer's instructions. The cDNA library was size-fractionated in a 1% agarose gel. 200-250 bp fragments were isolated and amplified by PCR with Illumina primers PE1 and PE2 for 15 cycles. The integrity and quality of total RNA and size-selected libraries was evaluated with Bioanalyzer 2100 (Agilent Technologies). Cluster formation, primer hybridization and single-end sequencing was performed as per the manufacturer's recommendations in the Weill Cornell Genomic Resources Core Facility on the Illumina Genome Analyzer IIx. Raw reads were mapped to the annotated mouse genome (Ensembl NCBIM37, Version 61.37.n, 36817 genes and 93809 transcripts) with the CLC Bio Genomic Workbench 4.8 software allowing up to 2 mismatches with the reference sequence. Multireads with more than 10 genomic matches were discarded. Low frequency multireads (2-10) were allocated using weighting function based on the distribution of unique reads at each paralog implemented in the CLC Bio Genomic workbench RNA-seq analysis module (see Table S1 for the summary of read distribution). The expression levels were normalized to the total exon length and the total number of mapped reads in a sample and expressed RPKM as in [1]. Out of 36,817 genes annotated in Ensembl NCBIM37, 10,338 genes with RPKM (reads per kilobase per million reads) > 1 in at least one experimental condition (U, D, L or L+D) were selected for further analysis. To exclude non-responsive genes, we filtered out all genes with the fold change of the mean group RPKM < 1.4 [2, 3]. The expression levels of 2,606 remaining genes were log2-transformed and compared using ANOVA with p-values corrected for multiple measurements using Benjamini-Hochberg false discovery rate (FDR) set to 0.1 [4]. 551 genes that passed FDR threshold were considered to be differentially expressed in at least one treatment condition.

ChIP-seq

ChIPs were performed as described in the main Experimental Procedures except cross-linking was performed in 1% methanol-free formaldehyde (Pierce). Chromatin was fragmented to 200-500 bp with Bioruptor (Diagenode; 17x30 sec cycles, high power at 4°C), cleared at 14,000 rpm at 4°C for 20 min and immunoprecipitated with 7 μg rabbit polyclonal anti-GR antibody (Santa Cruz Biotechnology, sc1004) and 40 μl of 50% protein A/G plus-agarose slurry per reaction at 4°C overnight. The quality of DNA shearing and library preparations was verified by Bioanalyzer (Agilent). The libraries were prepared and sequenced with HiSeq2000 by the Epigenomics Core Facility of Weill Cornell Medical College at average depth of 100 million reads/sample and converted to FASTQ files using the Illumina CASAVA 1.8.2 pipeline. The reads that have passed Illumina internal quality control were mapped to mouse genome as in RNA-seq section except that all multireads were discarded. Peaks were called using the Chipseq peak finder implemented in Genomics Workbench v.5.5. Read obtained from the untreated BMMΦ immunoprecipitated with anti-GR antibodies were used to calculate a background distribution with window size of 200 bp. Analyses were performed with several FDR cutoffs (1 to 5%) to optimize parameters and decrease the number of false-positive calls. Peaks were further filtered based on spatial distribution of forward and reverse reads orientation using the Wilcoxon rank test that tested whether the position of forward and reverse reads belonged to the same distribution with $p<10^{-4}$.

Gene association network construction and analysis

We included in the analysis co-expression data from GEO, interaction data from bioGRID, I2D interologus interaction database based on protein orthologies, molecular interactions from Pathways Commons and species-specific datasets and physical interactions [5]. The edges in

these networks correspond to functional links derived from gene association databases that are weighted according to the evidence of co-association [6]. We allowed the algorithm to find up to 20 genes that fit the overall network architecture based on the analysis of the composite network generated using either "equal by network" or GO-based network weighting. The gene list analysis was performed using the GeneMANIA Cytoscape plugin (ver. 3.1) within Cytoscape (ver. 2.8.3) network visualization and analysis environment (www.cytoscape.com).
Network partitioning was performed with Clustermaker Cytoscape plugin (ver. 1.10) using the Newman-Girvan community clustering algorithm. The nodes in resulting modules were overlaid with gene-specific parameters reflecting the response to Dex and/or LPS treatments using Multicolored Node plugin (ver. 2.540) [7] and the enrichment of genes with common parameters was tested using Yates corrected χ^2 test. Topological parameters were determined with a Cytoscape plugin NetworkAnalyzer [8] and compared to association networks generated from 10 sets of random genes that are not expressed in BMMΦ (referred to as "non-expressors").

Gene functional enrichment analysis

Gene ontology (GO) analysis was performed with BiNGO 2.44 Cytoscape plugin [9]. The significance of GO term enrichment was assessed by hypergeometric test corrected for multiple measurements using the Benjamini-Hochberg FDR correction with the significance level set to 0.05. The list of GO-enriched categories was further processed with EnrichmentMap 1.2 [10] to create networks of GO categories in which the nodes represent individual GO gene sets and the edges are weighted based on the degree of overlap between connected gene sets. Input GO gene sets were filtered for significance by setting the uncorrected p-values to 0.001 and FDR q to 0.1. The GO networks were generated with the Jaccard coefficient set to 0.37, arranged using the Cytoscape weighted force-directed layout and manually adjusted for clarity.

Modeling of expression data

The experiments were performed 3 times with BMMΦ prepared from 3 independent mice. BMMΦ were plated at 2 million per well in a 6-well plate one day prior to treatment. BMMΦ were treated with 100 nM Dex for up to 9 h and the expression levels of Dex-responsive genes were determined by qPCR (the expression level at t=0 was set to 1). The expression data were subjected to the global least-square fit to an equation describing the expression of a jointly regulated gene (Z) in the I-FFL under the control of a master TF (X) and an X-activated strong repressor (Y, Figure 4C). The non-linear least-square fitting was performed using the DataFit software (Oakdale Engineering) to a user-defined function [11]

(1) $$Z(t)=[e^{t}+ \ln(fe^{t}-f+1) - \frac{1}{f}\ln(fe^{t}-f+1)]e^{-t} ,$$

where f as a fitting parameter equal to the fold activity change of a master TF (X) and t is time. We used the coefficient of determination

$R^2 = 1 - \sum_{i=1}^{n}(Zi - \hat{Z}i)^2 \Big/ \sum_{i=1}^{n}(Zi - \bar{Z})^2$, where $\hat{Z}i$ is the ith predicted value of Z, that reflects the proportion of variation which is explained by the regression model as a goodness of fit measurement (Figure 4C). The accumulation of Y and Z over time is described by the following ordinary differential equations:

(2) $dY/dt = \beta1*X - \alpha1*Y$ and

(3) $d[Z]/dt = \beta2*X/Y - \alpha2*[Z]$,

where α1 and α2 are degradation rates for Y and Z, respectively, and β1 and β2 are maximal rate of production of Y and Z, respectively. Eq 2 and 3 were solved numerically using the Runge-Kutta-Fehlberg numerical integration algorithm as implemented in Polymath 6.1 ordinary differential equation solver (Polymath Software) and the goodness of fit to experimental data was evaluated by calculating R^2. The best numerical solution shown in Figure 4C for the

equations (2) and (3) was achieved with the following parameters: β1=β2=1, α1=1.2, α2=1; X=22 that were derived from the initial data fitting to equation (1).

Supplemental References

1. Mortazavi A, Williams BA, McCue K, Schaeffer L, Wold B: **Mapping and quantifying mammalian transcriptomes by RNA-Seq.** *Nat Methods* 2008, **5:**621-628.
2. Shi L, Jones WD, Jensen RV, Harris SC, Perkins RG, Goodsaid FM, Guo L, Croner LJ, Boysen C, Fang H, et al: **The balance of reproducibility, sensitivity, and specificity of lists of differentially expressed genes in microarray studies.** *BMC Bioinformatics* 2008, **9 Suppl 9:**S10.
3. Marioni JC, Mason CE, Mane SM, Stephens M, Gilad Y: **RNA-seq: an assessment of technical reproducibility and comparison with gene expression arrays.** *Genome Res* 2008, **18:**1509-1517.
4. Benjamini YaH, Y.: **Controlling the false discovery rate —a practical and powerful approach to multiple testing.** *J R Stat Soc Ser B, Stat Methodol* 1985, **57:**289-300.
5. Mostafavi S, Ray D, Warde-Farley D, Grouios C, Morris Q: **GeneMANIA: a real-time multiple association network integration algorithm for predicting gene function.** *Genome Biol* 2008, **9 Suppl 1:**S4.
6. Mostafavi S, Morris Q: **Fast integration of heterogeneous data sources for predicting gene function with limited annotation.** *Bioinformatics* 2010, **26:**1759-1765.
7. Warsow G, Greber B, Falk SS, Harder C, Siatkowski M, Schordan S, Som A, Endlich N, Scholer H, Repsilber D, et al: **ExprEssence--revealing the essence of differential experimental data in the context of an interaction/regulation net-work.** *BMC Syst Biol* 2010, **4:**164.
8. Assenov Y, Ramirez F, Schelhorn SE, Lengauer T, Albrecht M: **Computing topological parameters of biological networks.** *Bioinformatics* 2008, **24:**282-284.
9. Maere S, Heymans K, Kuiper M: **BiNGO: a Cytoscape plugin to assess overrepresentation of gene ontology categories in biological networks.** *Bioinformatics* 2005, **21:**3448-3449.
10. Merico D, Isserlin R, Stueker O, Emili A, Bader GD: **Enrichment map: a network-based method for gene-set enrichment visualization and interpretation.** *PLoS One* 2010, **5:**e13984.
11. Goentoro L, Shoval O, Kirschner MW, Alon U: **The incoherent feedforward loop can provide fold-change detection in gene regulation.** *Mol Cell* 2009, **36:**894-899.
