# Supplementary material for: Glucocorticoid receptor coordinates transcription factor-dominated regulatory network in macrophages
Source: BMC Genomics. 2014 Aug 6;15(1):656. doi: 10.1186/1471-2164-15-656 (PMC4133603; doi:10.1186/1471-2164-15-656)
Supplement: Supplementary file 2 — Additional file 2: Table S1: Contains statistical summary of combined RNA-seq experiments. Table S2. contains a detailed summary of genes regulated in BMMΦ upon a 1-h treatment with Dex (D), LPS (L) and LPS + Dex (L + D) compared to untreated control (U). Table S3. contains RT-qPCR and ChIP primers used in this study. (PDF 265 KB) [file 12864_2014_6351_MOESM2_ESM.pdf]

Table S1 Summary of RNA-seq experiments

	Untreated	%	Dex	%	LPS	%	LPS+Dex	%
Exon-exon reads	10,937,370	15.36	10,141,134	14.93	10,619,836	15.11	10,227,155	14.83
Exon-intron reads	184,875	0.26	180,216	0.27	182,942	0.26	171,746	0.25
Total exon reads	66,883,256	93.90	62,942,208	92.66	65,955,587	93.85	63,991,848	92.78
Total intron reads	4,341,916	6.10	4,988,485	7.34	4,325,522	6.15	4,980,262	7.22
Total gene reads	71,225,172	100.00	67,930,693	100.00	70,281,109	100.00	68,972,110	100.00

Table S2 Genes regulated in BMMΦ treated for 1 h with Dex (D), LPS (L) and LPS+Dex (L+D) compared to untreated control (U)

Unindexed expression values represent Log2 transformed average RPKMs. Expression values with z index were Z-transformed as described in Methods.

Cluster 1

Feature ID	Name	FDR, P	U	D	L	(L+D)	Uz	Dz	Lz	(L+D)z
Cited2	Cbp/p300-interacting transactivator, with Glu/Asp-rich carboxy-terminal dom	0.00523	5.57	8.31	7.5	9.54	-1.29618	0.348049	-0.13802	1.086152
Apbb3	amyloid beta (A4) precursor protein-binding, family B, member 3	0.00836	2.29	3.13	3.96	4.71	-1.17968	-0.37568	0.41875	1.136607
Plagl2	pleiomorphic adenoma gene-like 2	0.00855	4.24	4.84	5.16	5.6	-1.2582	-0.2097	0.349499	1.118398
Nfil3	nuclear factor, interleukin 3, regulated	0.00986	2.71	4.25	4.47	5.95	-1.23314	-0.07165	0.094277	1.210514
2010002N04Rik	RIKEN cDNA 2010002N04 gene	0.01000	2.86	3.47	4.2	5.33	-1.04034	-0.46604	0.221249	1.285127
Dusp1	dual specificity phosphatase 1	0.01000	2.9	6.24	8.79	9.39	-1.33015	-0.19969	0.663382	0.866459
Icosl	icos ligand	0.01000	2.54	4.43	5.44	5.3	-1.41354	0.001872	0.758257	0.653411
Chka	choline kinase alpha	0.02000	1.09	2.14	1.76	2.5	-1.29801	0.443728	-0.18661	1.040894
Mtmr12	myotubularin related protein 12	0.02000	2.95	3.32	3.74	4.13	-1.14398	-0.42044	0.400883	1.163539
Plaur	plasminogen activator, urokinase receptor	0.03000	4.27	5.39	6.62	6.93	-1.25712	-0.33838	0.670602	0.924898
Jdp2	Jun dimerization protein 2	0.04000	3.55	4.65	6.29	7.23	-1.14255	-0.47404	0.522657	1.093934
Zfp811	zinc finger protein 811	0.04000	0.64	1.14	1.6	1.97	-1.21146	-0.34303	0.455925	1.098561
Plscr1	phospholipid scramblase 1	0.05000	2.4	3.04	3.81	5.25	-0.99825	-0.47671	0.150756	1.324205
Cebpb	Cebpb	0.05000	6.97	7.99	8.92	9.46				
Med13	mediator complex subunit 13	0.06000	1.46	2.01	2.33	2.63	-1.2939	-0.19483	0.444622	1.044113
Prr7	proline rich 7 (synaptic)	0.06000	0.4	0.77	1.15	1.73	-1.07845	-0.42698	0.242102	1.263333
Adm	adrenomedullin	0.07000	0.51	1.36	2.29	2.4	-1.27528	-0.316	0.73357	0.857713
AI848100	expressed sequence AI848100	0.07000	2.23	2.61	3.05	3.83	-1.0186	-0.46565	0.174617	1.309631
Mocs1	molybdenum cofactor synthesis 1	0.08000	4.52	5.41	5.13	5.64	-1.35356	0.48563	-0.09299	0.960927
Sap30	sin3 associated polypeptide	0.08000	3.8	4.51	5.1	5.09	-1.34086	-0.18691	0.772009	0.755756
Tiparp	TCDD-inducible poly(ADP-ribose) polymerase	0.08000	2.52	4.05	4.35	5.08	-1.37379	0.046412	0.324883	1.002496
Zfand5	zinc finger, AN1-type domain 5	0.10000	3.91	4.57	5.25	5.82	-1.18041	-0.38341	0.437746	1.126065
5730508B09Rik	RIKEN cDNA 5730508B09 gene	0.11000	2.03	2.53	2.5	2.84	-1.33187	0.164614	0.074824	1.092436
	Cluster average		2.700	3.735	4.295	4.950	-1.230	-0.175	0.341	1.064
	Cluster SD		1.369	1.792	1.965	2.144	0.119	0.303	0.286	0.175

Cluster 2

Feature ID	Name	FDR, P	U	D	L	(L+D)	Uz	Dz	Lz	(L+D)z
Maff	v-maf musculoaponeurotic fibrosarcoma oncogene family, protein F (avian)	0.0023	1.18	1.12	6.39	6.18	-0.85552	-0.87575	0.901035	0.830233
Tnfaip3	tumor necrosis factor, alpha-induced protein 3	0.0023	2.21	2.58	8.2	8.27	-0.91994	-0.81032	0.854757	0.875496
Gramd1a	GRAM domain containing 1A	0.0023	4.88	4.87	6.24	6.27	-0.85964	-0.87219	0.847088	0.884736
Gm16854		0.0023	1.54	1.89	4.67	5.37	-0.94459	-0.76368	0.673229	1.03504
Tnfsf9	tumor necrosis factor (ligand) superfamily, member 9	0.0023	1.2	1.3	8.62	8.66	-0.8777	-0.85426	0.861292	0.870667
Nfkbia	nuclear factor of kappa light polypeptide gene enhancer in B-cells inhibitor, a	0.00256	5.9	7.35	9.99	9.99	-1.1854	-0.47145	0.828423	0.828423
Ccrl2	chemokine (C-C motif) receptor-like 2	0.00423	2.44	2.44	8.06	7.85	-0.86571	-0.86571	0.898676	0.832747
Errfi1	ERBB receptor feedback inhibitor 1	0.00428	2.05	3.16	6.81	6.37	-1.08321	-0.61123	0.940764	0.753674
Sdc4	syndecan 4	0.00477	5.29	6.17	9.76	10.26	-1.02979	-0.67854	0.75438	0.953952
Csf3	colony stimulating factor 3 (granulocyte)	0.00492	0	0	4.16	4.44	-0.86511	-0.86511	0.808776	0.921442
Cxcl1	chemokine (C-X-C motif) ligand 1	0.00592	1.12	0.78	9.05	9.56	-0.82967	-0.90006	0.81207	0.917655
Siah2	seven in absentia 2	0.0063	2.35	2.08	4.45	4.5	-0.75976	-0.96593	0.843757	0.881936
Gem	GTP binding protein (gene overexpressed in skeletal muscle)	0.00661	0.13	0.13	4.64	4.46	-0.86567	-0.86567	0.90092	0.830413
Sele	selectin, endothelial cell	0.00661	0.0073	0.02	1.47	2.6	-0.81046	-0.8003	0.355152	1.255606
Dpep3	dipeptidase 3	0.00731	0.55	0.76	2.05	2.12	-0.98731	-0.73446	0.818742	0.903025
Cxcl3	chemokine (C-X-C motif) ligand 3	0.00804	0.74	0.64	5.42	5.33	-0.84737	-0.88433	0.88248	0.849214
Ptger4	prostaglandin E receptor 4 (subtype EP4)	0.00855	2.18	2.45	4.34	4.31	-0.9779	-0.74629	0.874965	0.84923
Cd14	CD14 antigen	0.00918	6.74	7.25	9.83	10.4	-0.99278	-0.71382	0.697409	1.009192
Zc3h12c	zinc finger CCCH type containing 12C	0.00926	1.75	2.65	5.15	5.13	-1.10552	-0.58731	0.852171	0.840655
Kdm6b	KDM1 lysine (K)-specific demethylase 6B	0.00954	3.31	3.69	6.13	5.91	-0.98914	-0.72992	0.934564	0.784488
Itpkc	inositol 1,4,5-trisphosphate 3-kinase C	0.00974	1.83	1.79	4.55	5.52	-0.83654	-0.85755	0.592277	1.101819
Marcksl1	MARCKS-like 1	0.00974	6.02	5.85	10.14	9.99	-0.82975	-0.90099	0.896804	0.833944
Ets2	E26 avian leukemia oncogene 2, 3' domain	0.01	3.79	3.77	6.15	6.18	-0.85871	-0.87324	0.855083	0.876869
2310014H01Rik	RIKEN cDNA 2310014H01 gene	0.01	5.27	5.22	5.88	6.11	-0.78909	-0.90182	0.586185	1.104732
Ccl7	chemokine (C-C motif) ligand 7	0.01	1.78	1.14	6.48	6.92	-0.75611	-0.96651	0.788984	0.933631
Rabgef1	RAB guanine nucleotide exchange factor (GEF) 1	0.01	2.8	2.78	4.84	5.09	-0.85522	-0.8711	0.763947	0.962375
Bcl2a1c	B-cell leukemia/lymphoma 2 related protein A1c	0.01	0.04	0.09	2.23	2.82	-0.8711	-0.8364	0.648987	1.058509
Cflar	CASP8 and FADD-like apoptosis regulator	0.01	3.34	3.69	5.93	5.89	-0.98726	-0.7355	0.875765	0.846993
Hbegf	heparin-binding EGF-like growth factor	0.01	0.54	0.39	1.96	2.26	-0.779	-0.93532	0.700841	1.013484
Cxcl2	chemokine (C-X-C motif) ligand 2	0.02	1.11	0.6	8.88	8.87	-0.81014	-0.92017	0.86623	0.864072
Fosl2	fos-like antigen 2	0.02	3.57	4.08	5.27	5.45	-1.11954	-0.56114	0.741797	0.93888
9930022D16Rik	RIKEN cDNA 9930022D16 gene	0.02	0.43	0.63	1.62	2.12	-0.95738	-0.70871	0.522206	1.14388
Zc3h12a	zinc finger CCCH type containing 12A	0.02	4.1	4.77	6.59	6.94	-1.08682	-0.60138	0.717303	0.970896
Dusp2	dual specificity phosphatase 2	0.02	2.12	3.26	7.48	7.88	-1.04862	-0.65859	0.785181	0.922032
Vcam1	vascular cell adhesion molecule 1	0.02	0.98	1.24	3.92	3.78	-0.94546	-0.78158	0.907643	0.8194
Cd40	CD40 antigen	0.02	2.02	2.18	5.36	5.19	-0.90844	-0.82127	0.911165	0.81855
Eif1	eukaryotic translation initiation factor 1	0.02	6.3	6.28	6.82	7.14	-0.7989	-0.8466	0.441185	1.204317
Cxcl5	chemokine (C-X-C motif) ligand 5	0.02	0.19	0.15	0.87	1.45	-0.76749	-0.83213	0.331235	1.268386
Trim13	tripartite motif-containing 13	0.03	1.48	1.76	3.85	3.85	-0.97094	-0.75432	0.862632	0.862632
Ccrn4l	CCR4 carbon catabolite repression 4-like (S. cerevisiae)	0.03	1.04	1.14	4.47	4.41	-0.89161	-0.83992	0.881269	0.850257
Gm13822	predicted gene 13822	0.03	0.26	0.49	1.41	1.76	-1.00109	-0.6813	0.597876	1.084519
Slfn2	schlafen 2	0.03	7.24	7.85	9.31	9.59	-1.10942	-0.57125	0.716824	0.963853
Gbp5	guanylate binding protein 5	0.03	1.17	1.29	4.95	4.91	-0.89385	-0.83769	0.875132	0.856413
N4bp1	NEDD4 binding protein 1	0.03	3.95	4.38	5.39	5.7	-1.09616	-0.57533	0.648005	1.023485
Birc3	baculoviral IAP repeat-containing 3	0.03	4.17	4.55	6.15	5.99	-1.04342	-0.66399	0.933585	0.773827
Dnajb4	DnaJ (Hsp40) homolog, subfamily B, member 4	0.03	1.98	2.07	3.86	4.12	-0.90128	-0.82234	0.747781	0.975843
Sbds	Shwachman-Bodian-Diamond syndrome homolog (human)	0.04	4.24	4.22	5.32	5.28	-0.84947	-0.88183	0.898008	0.833287
Arf2	ADP-ribosylation factor 2	0.04	4.29	4.18	4.64	5.1	-0.63351	-0.89898	0.211171	1.321325
Ehd1	EH-domain containing 1	0.04	6.6	6.5	8.48	8.52	-0.82099	-0.90974	0.847615	0.883117
C5ar1	complement component 5a receptor 1	0.04	7.85	7.8	8.84	8.8	-0.82166	-0.9086	0.899909	0.830351

AC159314.1	Icam5 - predicted miRNA mmu-mir-1900	0.05	0.27	0.34	1.56	1.63	-0.91168	-0.81783	0.817829	0.911678
Ube2f	ubiquitin-conjugating enzyme E2F (putative)	0.05	3.64	3.7	4.51	4.71	-0.91014	-0.80093	0.673507	1.037565
Clec4e	C-type lectin domain family 4, member e	0.04	2.07	2.58	6.55	6.73	-0.9646	-0.76069	0.826659	0.898629
Snx20	sorting nexin 20	0.05	5.61	5.6	6.52	6.55	-0.85647	-0.87508	0.837847	0.893703
Irak2	interleukin-1 receptor-associated kinase 2	0.05	3.4	3.44	5.01	4.97	-0.8878	-0.84369	0.887802	0.843687
Gch1	GTP cyclohydrolase 1	0.05	2.5	2.53	4.4	4.32	-0.87964	-0.85149	0.903093	0.82803
Sema6d	sema domain, transmembrane domain (TM), and cytoplasmic domain, (sema	0.04	0.59	0.81	1.65	1.64	-1.05343	-0.65557	0.863544	0.845459
Mobkl2c	MOB1, Mps One Binder kinase activator-like 2C (yeast)	0.05	3.26	2.99	4.34	4.66	-0.68038	-1.01287	0.649591	1.043655
Fam177a	family with sequence similarity 177, member A	0.05	1.25	1.12	2.2	2.68	-0.74753	-0.92029	0.514964	1.152856
F3	coagulation factor III	0.04	0.09	0.16	1.69	2.85	-0.8351	-0.78232	0.371367	1.246058
Mia1	melanoma inhibitory activity 1	0.05	0.46	0.5	0.98	1.37	-0.85069	-0.7581	0.353008	1.255784
Plk2	polo-like kinase 2 (Drosophila)	0.04	3.59	2.49	5.54	6.51	-0.5166	-1.11953	0.552226	1.083898
Gm6377	predicted gene 6377	0.07	1.23	1.52	4.72	5.23	-0.92964	-0.79103	0.738452	0.982212
Rnf19b	ring finger protein 19B	0.06	5.62	5.92	7.8	8.11	-0.97547	-0.73994	0.736019	0.979396
Gtf2ird2	GTF2I repeat domain containing 2	0.07	1.81	1.97	3.62	3.64	-0.94364	-0.78471	0.854239	0.874105
Slc25a25	solute carrier family 25 (mitochondrial carrier, phosphate carrier), member 2	0.06	2.03	2.32	3.65	3.84	-1.01371	-0.69761	0.752106	0.959208
Slc25a33	solute carrier family 25, member 33	0.06	1.94	1.88	2.87	3.12	-0.80689	-0.90135	0.657317	1.050921
Cd69	CD69 antigen	0.06	1.09	0.98	4.5	5.65	-0.82573	-0.87196	0.60722	1.090474
Mex3c	mex3 homolog C (C. elegans)	0.06	3	3.21	4.31	4.24	-1.01245	-0.70431	0.909734	0.807022
Dusp16	dual specificity phosphatase 16	0.08	1.08	1.13	2.76	3.05	-0.88419	-0.8364	0.721693	0.998899
Igsf6	immunoglobulin superfamily, member 6	0.08	4.04	4.25	5.65	5.99	-0.96102	-0.7469	0.680618	1.027299
BC006779	cDNA sequence BC006779	0.08	2.46	2.68	4.11	4.19	-0.98124	-0.74138	0.817701	0.904922
Ankrd33b	ankyrin repeat domain 33B	0.08	2.54	2.47	3.72	3.79	-0.81625	-0.91309	0.816249	0.913092
Tlr2	toll-like receptor 2	0.08	4.75	4.59	7.75	7.8	-0.8208	-0.90999	0.85146	0.879331
1810029B16Rik	RIKEN cDNA 1810029B16 gene	0.08	1.21	1.26	2.08	2.36	-0.89159	-0.80545	0.607315	1.089721
Pde4b	phosphodiesterase 4B, cAMP specific	0.07	2.06	2.12	5.21	5.05	-0.88242	-0.84826	0.910886	0.819798
Gm15832	predicted gene 15832	0.09	1.83	1.94	2.66	3.42	-0.85838	-0.70909	0.26803	1.299438
Bcl2a1b	B-cell leukemia/lymphoma 2 related protein A1b	0.09	3.49	3.44	7.56	7.84	-0.85484	-0.87526	0.807857	0.922244
Gtf2b	general transcription factor IIB	0.1	4.38	4.39	5.35	5.37	-0.87479	-0.85703	0.848149	0.883674
Il10	interleukin 10	0.1	2.02	1.72	6.63	7.5	-0.80969	-0.90893	0.715403	1.003218
a	nonagouti	0.09	0.35	0.53	2.13	2.14	-0.95529	-0.77188	0.858492	0.868682
Tmem88	transmembrane protein 88	0.1	0.16	0.17	1.49	1.48	-0.87256	-0.85944	0.872561	0.85944
Cdk5r1	cyclin-dependent kinase 5, regulatory subunit 1 (p35)	0.09	0.5	0.41	1.98	1.94	-0.81337	-0.91683	0.888092	0.842107
Ier2	immediate early response 2	0.09	3.85	3.53	6.65	6.71	-0.77106	-0.95588	0.846143	0.880797
1700047I17Rik2	RIKEN cDNA 1700047I17 gene 2	0.09	1.31	1.02	2.19	2.67	-0.63693	-1.01582	0.512809	1.139939
Ccl12	chemokine (C-C motif) ligand 12	0.11	1	1.21	5.03	5.67	-0.9032	-0.81805	0.730873	0.990378
Ifrd1	interferon-related developmental regulator 1 (predicted miRNA mmu-mir-19	0.11	1.48	1.57	4.34	4.18	-0.89352	-0.83659	0.915658	0.814445
Rffl	ring finger and FYVE like domain containing protein	0.1	2.3	2.6	3.57	3.81	-1.05063	-0.64129	0.682227	1.009696
Ptx3	pentraxin related gene	0.11	0.09	0.12	1.46	2.91	-0.78795	-0.76554	0.235265	1.318229
	Cluster average		2.4766	2.5919	5.0091	5.2352	-0.89452	-0.8087	0.747048	0.956171
	Cluster SD		1.9137	1.9998	2.3709	2.3057	0.113799	0.112627	0.173338	0.136569

Cluster 3

Feature ID	Name	FDR, P	U	D	L	(L+D)	Uz	Dz	Lz	(L+D)z
Phlda1	pleckstrin homology-like domain, family A, member 1	0.0023	1.34	0.84	5.76	5.3	-0.76404	-0.95796	0.950203	0.771797
Nfkbiz	nuclear factor of kappa light polypeptide gene enhancer in B-cells inhibitor, z	0.0023	3.65	2.75	8.41	8.18	-0.70719	-1.01064	0.897691	0.820144
Socs3	suppressor of cytokine signaling 3	0.00291	2.8	2.27	8.77	8.55	-0.78936	-0.93891	0.895169	0.833093
Pim1	proviral integration site 1	0.00307	4.62	3.16	8.51	7.77	-0.54854	-1.12264	0.981085	0.690102
Dnajb5	DnaJ (Hsp40) homolog, subfamily B, member 5	0.00307	1.64	1.48	2.38	2.28	-0.676	-1.03062	0.964131	0.742491
Ccl4	chemokine (C-C motif) ligand 4	0.00423	3.71	2.26	10.3	9.88	-0.68164	-1.03119	0.907039	0.805788
Icam1	intercellular adhesion molecule 1	0.00423	5.03	4.43	8.46	8.14	-0.71403	-1.00252	0.935208	0.781343
Gadd45b	growth arrest and DNA-damage-inducible 45 beta	0.00477	4.4	3.92	8.04	7.73	-0.75003	-0.97192	0.932627	0.789324
2310016C08Rik	Hilpda, hypoxia inducible lipid droplet associated	0.00492	3.69	2.94	6.46	6.2	-0.63961	-1.06319	0.924818	0.777977
Tnf	tumor necrosis factor	0.00492	2.6	0.67	9.68	8.41	-0.62544	-1.06599	0.990663	0.700768
Ccl2	chemokine (C-C motif) ligand 2	0.00641	2.46	1.33	7.73	7.26	-0.68316	-1.02857	0.927698	0.784035
Rab20	RAB20, member RAS oncogene family	0.00729	4.89	4.43	7.15	7.17	-0.70076	-1.01679	0.851903	0.865644
Dusp5	dual specificity phosphatase 5	0.00747	2.79	1.71	6.93	6.63	-0.64969	-1.05645	0.909561	0.796572
Rasgef1b	RasGEF domain family, member 1B	0.0079	4.54	2.56	8.41	7.78	-0.46505	-1.18303	0.938262	0.709816
Nfkbie	nuclear factor of kappa light polypeptide gene enhancer in B-cells inhibitor, e	0.00908	3.19	2.7	6.29	5.95	-0.72599	-0.99098	0.950418	0.766553
Prdm1	PR domain containing 1, with ZNF domain	0.00954	2.72	1.58	5.43	4.68	-0.49935	-1.1444	1.034062	0.609686
Skil	SKI-like	0.01	3.27	2.88	5.44	4.98	-0.69412	-1.00438	1.032227	0.666274
Mapk6	mitogen-activated protein kinase 6	0.01	4.94	4.73	6.29	6.09	-0.72357	-0.98898	0.982663	0.729888
Ier5	represses cdc25B	0.01	4.27	3.02	5.81	5.47	-0.2939	-1.28016	0.921161	0.6529
4930430E12Rik	RIKEN cDNA 4930430E12 gene	0.02	3.52	3.03	5.05	4.44	-0.54035	-1.08069	1.146857	0.474181
Ccl3	chemokine (C-C motif) ligand 3	0.02	4.94	3.57	9.85	9.04	-0.62294	-1.06977	0.978447	0.714266
Cdc42ep2	CDC42 effector protein (Rho GTPase binding) 2	0.02	1.84	1.09	5.51	4.67	-0.67085	-1.02086	1.041865	0.649853
Junb	Jun-B oncogene	0.02	5.88	4.51	9.23	8.67	-0.52986	-1.1386	0.958644	0.709818
Bcl6	B-cell leukemia/lymphoma 6	0.02	3.19	2.49	4.58	4.17	-0.44129	-1.18117	1.027912	0.594551
Nod2	nucleotide-binding oligomerization domain containing 2	0.02	1.31	0.98	3.82	3.73	-0.75418	-0.97059	0.891898	0.832875
Cd83	CD83 antigen	0.02	4.37	3.66	8.42	7.61	-0.69973	-1.00175	1.023015	0.678465
Tnfaip2	tumor necrosis factor, alpha-induced protein 2	0.03	6.24	5.67	9.05	8.52	-0.67888	-1.02132	1.009305	0.690893
Irf1	interferon regulatory factor 1	0.03	4.66	3.52	7.48	6.46	-0.48982	-1.13164	1.097864	0.523597
Nfkbid	nuclear factor of kappa light polypeptide gene enhancer in B-cells inhibitor, d	0.03	3	2	5.88	5.29	-0.56562	-1.10818	0.996951	0.676841
3110043O21Rik	RIKEN cDNA 3110043O21 gene	0.03	3.48	3.17	4.69	4.51	-0.64288	-1.05592	0.969318	0.729487
Mtmr14	myotubularin related protein 14	0.03	4.34	4.21	5.35	5.22	-0.74838	-0.9695	0.969496	0.748383
Insig1	insulin induced gene 1	0.03	4.4	3.98	5.35	5.06	-0.47833	-1.15362	1.049115	0.582842
2210403K04Rik	mir22	0.04	3.43	3.05	5.2	4.88	-0.67055	-1.02943	1.001099	0.698881
Pvr	poliovirus receptor	0.04	1.9	1.6	4.08	4.09	-0.75166	-0.97328	0.858776	0.866163
Batf	basic leucine zipper transcription factor, ATF-like	0.05	4.25	3.97	4.96	4.85	-0.54195	-1.13124	0.95235	0.72084
Tmem171	transmembrane protein 171	0.04	2.28	2	3.56	3.26	-0.65813	-1.03041	1.043702	0.644835
Adora2b	adenosine A2b receptor	0.07	1.96	1.2	5.11	4.75	-0.65925	-1.04614	0.944329	0.761063
Lcp2	lymphocyte cytosolic protein 2	0.06	4.77	4.63	6.33	6.25	-0.78775	-0.93987	0.907269	0.820345

Tgif1	TGFB-induced factor homeobox 1	0.07	4.19	3.14	5.56	5.12	-0.29125	-1.26986	0.985599	0.575515
Gm10941	predicted gene 10941	0.08	0.64	0.42	1.39	1.35	-0.62818	-1.07398	0.891607	0.810552
Rhbdf2	rhomboid 5 homolog 2 (Drosophila)	0.09	3.93	3.59	5.58	5.33	-0.68182	-1.02399	0.978706	0.727111
Egr1	early growth response 1	0.09	2.34	1.88	6.9	6.61	-0.7776	-0.94854	0.916957	0.809189
Hpn	hepsin	0.1	2.14	1.65	3.37	2.92	-0.4928	-1.12825	1.102317	0.518737
	Cluster average		3.4779	2.7598	6.3384	5.936	-0.63106	-1.05553	0.96907	0.717523
	Cluster SD		1.2848	1.2684	2.0529	1.9341	0.121267	0.081904	0.064721	0.094224

Cluster 4

Feature ID	Name	FDR, P	U	D	L	(L+D)	Uz	Dz	Lz	(L+D)z
Il1b	interleukin 1 beta	0.00423	1.46	0.5	9.39	7.51	-0.7401	-0.95838	1.062968	0.635507
Csrnp1	cysteine-serine-rich nuclear protein 1	0.00423	2.43	2.33	7.41	6.76	-0.84351	-0.88014	0.980888	0.742764
Mdm2	transformed mouse 3T3 cell double minute 2	0.00423	4.06	4.03	5.93	5.36	-0.82398	-0.85547	1.138871	0.54057
Egr3	early growth response 3	0.00423	0.81	0.64	5.01	4.61	-0.82765	-0.89952	0.948146	0.779023
Ankrd57	ankyrin repeat domain 57	0.00423	2.11	2.02	4.3	4.1	-0.82734	-0.90016	0.944663	0.782836
BC031781	cDNA sequence BC031781	0.00436	3.26	3.18	5.29	5.08	-0.82806	-0.89835	0.955459	0.770956
Il1a	interleukin 1 alpha	0.00492	0.14	0.03	6.35	4.43	-0.82151	-0.8563	1.14252	0.535284
Gpr132	G protein-coupled receptor 132	0.00508	3.79	3.49	6.21	5.81	-0.74812	-0.96497	1.001112	0.711982
Psd	pleckstrin and Sec7 domain containing	0.00539	1.42	1.32	3.35	2.87	-0.80052	-0.89814	1.083628	0.615032
Irg1	immunoresponsive gene 1	0.0063	2.34	2.15	8.78	8.1	-0.83675	-0.8897	0.95798	0.768474
Nlrp3	NLR family, pyrin domain containing 3	0.00729	2.94	2.71	7.38	7.04	-0.81881	-0.90946	0.931137	0.797132
Ptgs2	prostaglandin-endoperoxide synthase 2	0.00855	0.54	0.25	6.2	4.65	-0.79675	-0.89425	1.106042	0.584958
Rel	reticuloendotheliosis oncogene	0.00855	2.3	2.52	5.2	4.83	-0.93282	-0.78753	0.982348	0.737999
Nupr1	nuclear protein 1	0.00908	1.78	1.76	5.43	5.06	-0.8586	-0.86854	0.955523	0.771625
CT027693.1	mIR-155	0.01	0.12	0.05	4.05	3.12	-0.83403	-0.86807	1.077186	0.624914
Lilrb4	leukocyte immunoglobulin-like receptor, subfamily B, member 4	0.01	7.29	7.22	8.43	8.22	-0.80085	-0.91296	1.025083	0.688728
Sod2	superoxide dismutase 2, mitochondrial	0.01	4.4	4.31	6.92	6.56	-0.82835	-0.89332	0.990768	0.730895
Cdkn1a	cyclin-dependent kinase inhibitor 1A (P21)	0.01	6.02	6.17	8.74	8.23	-0.9092	-0.80182	1.038065	0.672952
Mefv	Mediterranean fever	0.01	3.67	3.41	6.09	5.69	-0.76239	-0.95208	1.003151	0.711325
Bcl2a1a	B-cell leukemia/lymphoma 2 related protein A1a	0.01	0.16	0.28	3.75	3.37	-0.89396	-0.83196	0.961141	0.764779
Cxcl10	chemokine (C-X-C motif) ligand 10	0.01	2.2	1.66	9.07	8.43	-0.79447	-0.93109	0.943744	0.781815
Tpbg	trophoblast glycoprotein	0.01	0.26	0.27	1.81	1.19	-0.82273	-0.80951	1.225827	0.406406
Ifnb1	interferon beta 1, fibroblast	0.02	0.02	0	5.72	4.51	-0.85076	-0.85746	1.056553	0.651666
Gpr84	G protein-coupled receptor 84	0.02	1.76	1.38	6.17	4.93	-0.76334	-0.92449	1.10684	0.580985
Tnip3	TNFAIP3 interacting protein 3	0.02	2.65	2.31	5.52	4.84	-0.7424	-0.95631	1.063265	0.635442
Ripk2	receptor (TNFRSF)-interacting serine-threonine kinase 2	0.02	2.14	1.9	4.17	3.75	-0.74736	-0.95838	1.037512	0.668228
Fam55c	family with sequence similarity 55, member C	0.02	0.4	0.39	1.43	1.12	-0.83077	-0.84987	1.136344	0.544299
Trib1	tribbles homolog 1 (Drosophila)	0.02	3.11	2.79	5.39	4.82	-0.72103	-0.9725	1.070737	0.622796
Snx18	sorting nexin 18	0.02	4.31	4.39	6.22	5.96	-0.90036	-0.82121	0.989404	0.732159
Slc2a6	solute carrier family 2 (facilitated glucose transporter), member 6	0.02	2.87	2.57	5.81	4.88	-0.74178	-0.93321	1.134209	0.540783
1110002J07Rik	RIKEN cDNA 1110002J07 gene	0.02	0.63	0.65	2.97	2.78	-0.87218	-0.85671	0.937935	0.79096
Ppp1r10	protein phosphatase 1, regulatory subunit 10(Mir 1894)	0.02	3.4	3.34	4.44	4.37	-0.81421	-0.91442	0.922768	0.805857
Zfp131 (NT_110857	Zfp131	0.02	1.6	1.69	3.71	3	-0.87413	-0.78672	1.175219	0.485628
Gm2449	predicted gene 2449	0.02	0.12	0.29	1.59	1.42	-0.97101	-0.74642	0.971008	0.746421
Areg	amphiregulin	0.03	0.05	0	2.65	2.21	-0.84092	-0.87663	1.015892	0.701662
Gp49a	glycoprotein 49 A	0.03	3.74	3.82	5.28	4.96	-0.90413	-0.80226	1.056946	0.649449
Myc	myelocytomatosis oncogene	0.03	2.2	2.01	5.03	4.6	-0.79932	-0.91985	0.995974	0.723191
Clcf1	cardiotrophin-like cytokine factor 1	0.03	0.86	0.56	3.53	2.75	-0.73761	-0.94539	1.111612	0.571389
Gja1	gap junction protein, alpha 1	0.03	1.88	1.93	4.28	3.52	-0.85705	-0.81514	1.154608	0.517583
Ppp1r15a	protein phosphatase 1, regulatory (inhibitor) subunit 15A	0.03	2.85	2.83	7.74	7.45	-0.86158	-0.86886	0.917986	0.812449
Ccl5	chemokine (C-C motif) ligand 5	0.03	2.87	3.2	8.08	7.68	-0.92237	-0.80474	0.93485	0.792261
Mapkapk2	MAP kinase-activated protein kinase 2	0.03	6.8	6.63	8.54	7.87	-0.729	-0.91677	1.192903	0.452861
Ncrna00085	non-protein coding RNA 85, mir-125a	0.03	1.16	1.34	2.61	2.19	-0.96424	-0.70324	1.138239	0.529245
2500002B13Rik	RIKEN cDNA 2500002B13 gene	0.04	0.52	0.48	2.08	1.85	-0.83708	-0.88408	0.995687	0.725471
Ch25h	cholesterol 25-hydroxylase	0.04	0.25	0.14	2.76	1.93	-0.79223	-0.87767	1.157278	0.51262
Mmd	monocyte to macrophage differentiation-associated	0.04	3.66	3.61	4.84	4.74	-0.8266	-0.90141	0.938809	0.789198
AC124475.1		0.04	0.52	0.54	2.62	2.44	-0.8729	-0.85561	0.942038	0.786472
Malt1	mucosa associated lymphoid tissue lymphoma translocation gene 1	0.04	1.98	1.97	4.26	3.44	-0.82295	-0.83178	1.189196	0.465529
Rnf19a	ring finger protein 19A	0.04	3.42	3.19	4.67	4.19	-0.65338	-0.98919	1.171699	0.47087
Plek	pleckstrin	0.05	5.8	5.63	8.22	7.83	-0.79557	-0.92197	1.003761	0.713786
Il1f9	interleukin 1 family, member 9	0.05	1.19	1	3.01	2.27	-0.71696	-0.91802	1.20904	0.425942
Traf1	TNF receptor-associated factor 1	0.06	1.5	1.42	4.48	3.91	-0.83154	-0.88165	1.035115	0.678071
Rela	v-rel reticuloendotheliosis viral oncogene homolog A (avian)	0.06	5.37	5.27	6.43	6.11	-0.75181	-0.92871	1.123293	0.557224
Rab11fip1	RAB11 family interacting protein 1 (class I)	0.06	1.55	1.39	4.17	3.34	-0.77917	-0.8965	1.14217	0.533502
Oasl1	2'-5' oligoadenylate synthetase-like 1	0.06	2.16	2.07	5.05	4.74	-0.83518	-0.89106	0.959368	0.766874
Il6	interleukin 6	0.06	0.07	0.02	3.91	3.55	-0.8522	-0.87565	0.948327	0.779527
Tnfsf4	tumor necrosis factor (ligand) superfamily, member 4	0.06	0.35	0.28	2.93	1.87	-0.78741	-0.84212	1.228981	0.400542
Pip5k1a	phosphatidylinositol-4-phosphate 5-kinase, type 1 alpha	0.06	2.78	2.64	3.54	3.24	-0.65027	-0.98745	1.180123	0.457599
H2-Q7	histocompatibility 2, Q region locus 7	0.07	3.62	3.52	5.46	5.06	-0.80292	-0.90392	1.055412	0.651427
Tmem200b	transmembrane protein 200B	0.07	0.02	0.03	2.6	2.05	-0.85761	-0.85019	1.058094	0.649707
Orm2	orosomucoid 2	0.07	0.3	0.34	1.12	0.9	-0.89308	-0.79521	1.113294	0.574998
Trem1	triggering receptor expressed on myeloid cells 1	0.07	0.88	0.76	2.55	2.35	-0.79819	-0.92506	0.967347	0.755905
Nr4a1	nuclear receptor subfamily 4, group A, member 1	0.07	2.87	3.25	6.73	6.32	-0.95481	-0.76608	0.962258	0.758631
Hspa1a	heat shock protein 1A	0.07	0.75	0.83	3.75	2.65	-0.85131	-0.79661	1.200039	0.447878
Osgin2	oxidative stress induced growth inhibitor family member 2	0.08	0.79	0.78	1.8	1.58	-0.84405	-0.86291	1.060959	0.646006
Tnip1	TNFAIP3 interacting protein 1	0.08	4.84	4.76	6.67	6.34	-0.81733	-0.8978	1.023544	0.691584
Ifitm1	interferon induced transmembrane protein 1	0.08	3.23	3.16	4.08	3.92	-0.78158	-0.93045	1.026151	0.685873
Dyrk2	dual-specificity tyrosine-(Y)-phosphorylation regulated kinase 2	0.08	3.67	3.5	5.21	4.95	-0.75939	-0.95426	1.005839	0.707813
H2-Q8		0.08	1.65	1.41	3.46	3.12	-0.73769	-0.97065	1.019177	0.689158
Cdc42ep4	CDC42 effector protein (Rho GTPase binding) 4	0.08	2.92	2.57	5.9	4.98	-0.72961	-0.94741	1.124757	0.552267
Dcun1d3	DCN1, defective in cullin neddylation 1, domain containing 3 (S. cerevisiae)	0.08	1.26	1.46	2.36	2.05	-1.02335	-0.63163	1.131067	0.523914
Rnd1	Rho family GTPase 1	0.09	0.78	0.77	4.51	3.79	-0.8538	-0.85887	1.03902	0.67365

Atrip	ATR interacting protein	0.09	4.44	4.26	6.02	5.6	-0.74132	-0.94981	1.088811	0.602321
Zswim4	zinc finger, SWIM domain containing 4	0.09	3.57	3.22	6.24	5.67	-0.73518	-0.96804	1.04123	0.661996
Gm16912		0.09	0.2	0.01	1.63	1.2	-0.71767	-0.96117	1.114957	0.563886
Baz1a	bromodomain adjacent to zinc finger domain 1A	0.1	2.48	2.46	3.6	3.33	-0.83333	-0.86752	1.081197	0.619658
Arl5b	ADP-ribosylation factor-like 5B	0.1	1.02	1.01	2.36	2.04	-0.84346	-0.85782	1.080351	0.620932
Stx11	syntaxin 11	0.1	2.4	2.25	4.62	4.13	-0.79041	-0.91521	1.056647	0.648964
Klf6	Kruppel-like factor 6	0.1	5.13	5.22	7.58	7.3	-0.89667	-0.82813	0.969008	0.755788
Adora2a	adenosine A2a receptor	0.1	0.88	0.79	3.51	3.37	-0.83525	-0.89503	0.911638	0.818648
RP24-143P3.1		0.1	3.36	3.21	5.09	4.41	-0.73685	-0.90495	1.201931	0.439867
Zfp263	zinc finger protein 263	0.1	3.84	3.59	5.42	4.82	-0.67664	-0.96956	1.174605	0.4716
Nfkb2	nuclear factor of kappa light polypeptide gene enhancer in B-cells 2, p49/p1(	0.11	5.14	4.95	6.72	6.38	-0.74433	-0.95943	1.04433	0.659428
Spata13	spermatogenesis associated 13	0.11	1.3	1.28	2.87	2.38	-0.82565	-0.85077	1.145866	0.530552
Kctd12	potassium channel tetramerisation domain containing 12	0.11	5.89	5.88	7.41	6.9	-0.82652	-0.83964	1.167621	0.498535
AC102815.1		0.11	5.92	5.87	7.42	6.9	-0.79845	-0.86417	1.173031	0.489584
Zfp36	zinc finger protein 36	0.11	6.1	6.11	9.33	9.08	-0.86741	-0.86183	0.934346	0.794891
Il27	interleukin 27	0.11	0.73	0.98	3.76	3.5	-0.94008	-0.7847	0.943191	0.78159
Itga5	integrin alpha 5 (fibronectin receptor alpha)	0.11	4.31	5.02	7.47	6.83	-1.07443	-0.5969	1.050887	0.620444
	Cluster average		2.4082	2.3283	5.0134	4.4942	-0.82011	-0.87799	1.053579	0.644517
	Cluster SD		1.8186	1.8264	2.0545	2.0082	0.074362	0.070774	0.087638	0.115168

Cluster 5

Feature ID	Name	FDR, P	U	D	L	(L+D)	Uz	Dz	Lz	(L+D)z
Csf2	colony stimulating factor 2 (granulocyte-macrophage)	0.00291	0	0	1.12	0.1	-0.55925	-0.55925	1.494386	-0.37589
Arih2	ariadne homolog 2 (Drosophila)	0.00423	3.75	3.65	4.69	4.25	-0.69616	-0.90396	1.257238	0.342883
Dusp8	dual specificity phosphatase 8	0.00492	0.23	0.1	1.63	1.04	-0.72311	-0.90388	1.223718	0.403271
Ralgds	ral guanine nucleotide dissociation stimulator	0.00589	4.34	3.8	5.64	4.82	-0.39717	-1.08903	1.268397	0.217806
Etv3	ets variant gene 3	0.00661	3.49	2.73	5.08	4.13	-0.36904	-1.13221	1.22761	0.273639
Stk35	serine/threonine kinase 35	0.01	2.51	2.31	3.36	2.96	-0.58519	-1.01079	1.223589	0.372397
Irgq	immunity-related GTPase family, Q	0.01	2.33	2.32	3.45	2.95	-0.79376	-0.81211	1.261751	0.344114
Brd2	bromodomain containing 2	0.01	4.84	4.53	5.6	5.09	-0.38692	-1.07232	1.293417	0.165823
Sqstm1	sequestosome 1	0.02	7.47	7.15	8.97	8.14	-0.5743	-0.97165	1.28829	0.257658
Niacr1	niacin receptor 1	0.02	0.79	0.48	3.85	2.27	-0.68371	-0.88414	1.294689	0.273162
Olr1	oxidized low density lipoprotein (lectin-like) receptor 1	0.02	0.26	0.17	3.63	1.83	-0.74487	-0.80016	1.325402	0.219621
Il12b	interleukin 12b	0.02	0.14	0.11	3.34	1.56	-0.75174	-0.77139	1.344611	0.178517
Lif	leukemia inhibitory factor	0.02	0.25	0.1	2.84	1.08	-0.64994	-0.76919	1.409193	0.009938
Serpinb2	serine (or cysteine) peptidase inhibitor, clade B, member 2	0.02	1.23	1.27	3.8	2.58	-0.80756	-0.77493	1.288834	0.293658
Il1rn	interleukin 1 receptor antagonist	0.02	3.83	3.92	6.01	5.03	-0.84123	-0.75396	1.272758	0.322432
Tmem39a	transmembrane protein 39a	0.02	1.6	1.58	2.64	1.9	-0.66607	-0.70643	1.433052	-0.06055
Mxd1	MAX dimerization protein 1	0.03	2.96	2.59	5.48	4.11	-0.63354	-0.91767	1.301627	0.249575
Mybpc3	myosin binding protein C, cardiac	0.03	0.31	0.32	1.25	0.85	-0.81921	-0.79722	1.248057	0.368369
Orai2	ORAI calcium release-activated calcium modulator 2	0.04	3.53	3.14	5.48	4.36	-0.57713	-0.95384	1.306397	0.224575
Ets1	E26 avian leukemia oncogene 1, 5' domain	0.04	1.54	1.29	2.58	1.87	-0.50037	-0.94713	1.358154	0.089352
Vasp	vasodilator-stimulated phosphoprotein	0.04	6.2	6.11	6.96	6.62	-0.69209	-0.92068	1.238151	0.37462
Med21	mediator complex subunit 21	0.05	3.23	3.01	4.22	3.49	-0.48929	-0.90733	1.391876	0.00475
Vash1	vasohibin 1	0.05	0.76	0.68	2.22	1.24	-0.65684	-0.76985	1.405504	0.021188
Foxn2	forkhead box N2	0.06	2.57	2.33	3.65	2.91	-0.51314	-0.93061	1.365476	0.078276
Ccr3	chemokine (C-C motif) receptor 3	0.06	1.38	1.31	2.29	1.64	-0.61589	-0.77267	1.422157	-0.03359
Zfp295	zinc finger protein 295	0.06	2.09	1.81	2.71	2.31	-0.36859	-1.10577	1.263741	0.210624
Arl5c	ADP-ribosylation factor-like 5C	0.07	4.3	3.91	6.92	5.59	-0.64507	-0.93096	1.275487	0.300546
Il17ra	interleukin 17 receptor A	0.07	4.12	3.83	5.24	4.61	-0.53463	-1.00445	1.279866	0.259213
Fam43a	family with sequence similarity 43, member A	0.07	3.36	3.11	4.88	4.16	-0.64325	-0.95401	1.246111	0.351148
Cass4	Cas scaffolding protein family member 4	0.07	0.44	0.2	1.06	0.78	-0.47655	-1.11196	1.164907	0.423603
Spata2	spermatogenesis associated 2	0.08	3.33	3.07	4.16	3.62	-0.45988	-1.01602	1.315477	0.160424
Stk40	serine/threonine kinase 40	0.08	4.05	3.46	5.62	4.74	-0.44917	-1.08392	1.239922	0.29317
Mmp13	matrix metallopeptidase 13	0.08	2.46	2.38	4.73	3.1	-0.64887	-0.72224	1.433009	-0.06191
Nr4a3	nuclear receptor subfamily 4, group A, member 3	0.08	0.17	0.13	1.07	0.4	-0.62655	-0.71852	1.442783	-0.09772
Mrpl23-ps1	mitichondrial ribosomal protein L23, pseudogene 1	0.08	1.52	1.62	3.14	2.11	-0.77906	-0.64416	1.406357	0.016863
Klf7	Kruppel-like factor 7 (ubiquitous)	0.09	2	1.8	3.57	2.76	-0.66078	-0.90896	1.28743	0.282304
Hspa1b	heat shock protein 1B	0.1	1.41	1.19	4.95	3.28	-0.73524	-0.8599	1.270729	0.324411
Asprv1	aspartic peptidase, retroviral-like 1	0.1	1.47	1.39	2.9	2.25	-0.74675	-0.85894	1.258614	0.347083
Plekhg2	pleckstrin homology domain containing, family G (with RhoGef domain) mer	0.1	2.29	2.27	3.12	2.75	-0.77953	-0.82863	1.258292	0.349867
AI504432	expressed sequence AI504432, KCNA3 associate	0.1	0.3	0.26	1.65	0.67	-0.64926	-0.71109	1.437648	-0.07729
Rap2b	RAP2B, member of RAS oncogene family	0.1	5.41	5.01	6.54	6.01	-0.49481	-1.09006	1.18679	0.398077
Bcl2l11	BCL2-like 11 (apoptosis facilitator)	0.11	4.31	3.9	5.53	5	-0.51836	-1.08509	1.168029	0.435419
G0s2	G0/G1 switch gene 2	0.11	1.38	1.13	3.3	1.26	-0.3774	-0.62089	1.492567	-0.49428
	Cluster average		2.4174	2.2202	3.9737	3.0749	-0.61212	-0.88577	1.310979	0.18691
	Cluster SD		1.802	1.7166	1.79	1.8173	0.133502	0.145779	0.084979	0.207125

Cluster 6

Feature ID	Name	FDR, P	U	D	L	(L+D)	Uz	Dz	Lz	(L+D)z
Fbxl22	F-box and leucine-rich repeat protein 22	0.00423	0.09	0.25	0.51	1.06	-0.91143	-0.5351	0.076442	1.370083
Pfkfb3	6-phosphofructo-2-kinase/fructose-2,6-biphosphatase 3	0.00492	4.18	5.91	4.51	7.44	-0.89305	0.268587	-0.67147	1.295935
D10Wsu102e	DNA segment, Chr 10, Wayne State University 102, expressed	0.00954	3.16	3.59	3.12	5.18	-0.622	-0.17808	-0.66329	1.46337
Hspa14 (2 3406121.		0.01	2.32	2.74	2.47	3.35	-0.88002	0.044001	-0.55002	1.386039
Slc10a6	solute carrier family 10 (sodium/bile acid cotransporter family), member 6	0.02	0.05	0.28	0.03	1.12	-0.62414	-0.17554	-0.66315	1.462829
Htr2a	5-hydroxytryptamine (serotonin) receptor 2A	0.02	0.11	0.73	0.28	1.27	-0.93919	0.255268	-0.61168	1.295607
Mt2	metallothionein 2	0.02	6.87	7.81	7.78	10.41	-0.88339	-0.26715	-0.28682	1.437355
Glipr1	GLI pathogenesis-related 1 (glioma)	0.02	7.4	7.44	7.9	8.87	-0.73494	-0.67643	-0.00366	1.415025
Steap4	STEAP family member 4	0.03	0.09	0.09	0.56	1.71	-0.68347	-0.68347	-0.06867	1.435622
Pik3r1	phosphatidylinositol 3-kinase, regulatory subunit, polypeptide 1 (p85 alpha)	0.03	4.02	4.76	4.35	5.21	-1.09707	0.3398	-0.4563	1.213573
Rnf103	ring finger protein 103	0.04	2.88	3.75	3.59	4.9	-1.07535	-0.03584	-0.22702	1.33821
Rnf149	ring finger protein 149	0.05	5.89	6.29	6.32	6.99	-1.05823	-0.18094	-0.11514	1.354311

Hs6st1	heparan sulfate 6-O-sulfotransferase 1	0.06	3.9	4.08	3.88	4.79	-0.61347	-0.19281	-0.66021	1.466491
Saa1	serum amyloid A 1	0.07	0	0.03	0.06	1	-0.56114	-0.49936	-0.43759	1.498091
6330409N04Rik	RIKEN cDNA 6330409N04 gene	0.06	1.93	3.02	2.89	4.14	-1.17684	0.027625	-0.11603	1.265238
Edn1	endothelin 1	0.06	0.08	0.1	0.54	2.06	-0.65815	-0.63675	-0.16588	1.460778
S100a10	S100 calcium binding protein A10 (calpactin)	0.08	5.83	6.12	6.03	6.94	-0.81866	-0.22513	-0.40933	1.453121
Prdx6	peroxiredoxin 6	0.08	4.79	5.13	4.87	6.37	-0.68075	-0.21784	-0.57183	1.47042
Arc	activity regulated cytoskeletal-associated protein	0.08	0.54	0.86	1.04	2.21	-0.85473	-0.41535	-0.1682	1.438275
Gm16556	predicted gene 16556	0.09	0.93	1.24	1.12	1.92	-0.86428	-0.14501	-0.42344	1.432725
Map3k6	mitogen-activated protein kinase kinase kinase 6	0.09	0.82	2.13	1.59	3.27	-1.09976	0.172369	-0.35202	1.279413
Med7	mediator complex subunit 7	0.11	1.79	2.04	2.16	2.99	-0.87495	-0.39421	-0.16345	1.432604
Glrx	glutaredoxin	0.11	6.84	7.1	7.18	7.75	-0.98429	-0.30637	-0.09778	1.388435
	Cluster average		2.8048	3.2822	3.1643	4.3891	-0.85171	-0.20251	-0.33941	1.393633
	Cluster SD		2.5292	2.6268	2.5768	2.7521	0.179467	0.29801	0.238759	0.078437

Cluster 7

Feature ID	Name	FDR, P	U	D	L	(L+D)	Uz	Dz	Lz	(L+D)z
Cytip	cytohesin 1 interacting protein	0.04	2.78	4.05	2.72	4.21	-0.82521	0.762699	-0.90023	0.962751
Adrb2	adrenergic receptor, beta 2	0.03	2.97	4.4	2.97	4.71	-0.85786	0.690077	-0.85786	1.025644
Zfp217	zinc finger protein 217	0.02	4.15	4.71	4.06	4.88	-0.73885	0.640335	-0.9605	1.059015
Il15ra	interleukin 15 receptor, alpha chain	0.02	0.71	2.46	0.88	2.58	-0.94793	0.802865	-0.77785	0.922919
Fkbp5	FK506 binding protein 5	0.05	1.64	4.31	1.61	3.98	-0.85204	0.975227	-0.87257	0.749385
Tm6sf1	transmembrane 6 superfamily member 1	0.08	4.78	5.36	4.71	5.57	-0.76398	0.599433	-0.92853	1.093084
B4galt1	UDP-Gal:betaGlcNAc beta 1,4- galactosyltransferase, polypeptide 1	0.06	5.28	6.16	5.07	6.04	-0.65826	0.962068	-1.04493	0.741115
Btbd1	BTB (POZ) domain containing 1	0.06	4.44	5.01	4.19	5.12	-0.55911	0.715661	-1.11822	0.96167
1700029F09Rik	RIKEN cDNA 1700029F09 gene	0.09	0.96	2.59	1.21	2.61	-1.00209	0.848797	-0.71821	0.871508
Per1	period homolog 1 (Drosophila)	0.08	2.54	5.35	2.97	5.34	-1.00291	0.863436	-0.71732	0.856795
Kcnk6	potassium inwardly-rectifying channel, subfamily K, member 6	0.04	3.46	4.77	3.27	4.49	-0.72313	1.039287	-0.97875	0.662588
E130208F15Rik	RIKEN cDNA E130208F15 gene	0.11	2.61	3.17	2.42	3.43	-0.63043	0.556259	-1.03305	1.107221
Klf9	Kruppel-like factor 9 (mir1192)	0.06	1.51	3.3	1.28	2.87	-0.73306	1.064444	-0.96403	0.632642
Prss16	protease, serine, 16 (thymus)	0.02	0.28	1.96	0.4	2.64	-0.89179	0.548795	-0.78889	1.131891
Klf4	Kruppel-like factor 4 (gut)	0.04	1.49	4.32	1.69	3.79	-0.92417	1.038606	-0.78546	0.671019
Tigd2	tigger transposable element derived 2	0.08	2.47	4.83	2.25	4.23	-0.76188	1.082262	-0.93379	0.613412
Gpr31c	G protein-coupled receptor 31, D17Leh66c region	0.03	2.09	2.97	2.18	3.33	-0.91281	0.541076	-0.76411	1.135847
Ncoa5	nuclear receptor coactivator 5	0.04	2.93	3.87	3.12	3.83	-1.05102	0.895696	-0.65753	0.812857
Lyve1	lymphatic vessel endothelial hyaluronan receptor 1	0.08	0.04	1.03	0.04	1.54	-0.83183	0.491082	-0.83183	1.172583
Gm11707	predicted gene 11707	0.06	0.33	0.82	0.12	1.11	-0.58686	0.498281	-1.05193	1.140509
Tgfb3	transforming growth factor, beta 3	0.03	0.77	2.5	0.65	2.01	-0.77829	1.111447	-0.90937	0.576205
Dnajb13	DnaJ (Hsp40) related, subfamily B, member 13	0.11	1.15	2.42	0.69	2.09	-0.5437	1.034587	-1.11537	0.624481
Pex11a	peroxisomal biogenesis factor 11 alpha	0.02	1.64	3.14	1.4	2.67	-0.6913	1.119972	-0.98111	0.552439
Mmp19	matrix metallopeptidase 19	0.03	4.04	4.86	3.58	4.71	-0.43164	0.942896	-1.20272	0.691457
Thbd	thrombomodulin	0.08	2.25	3.15	1.61	3.1	-0.376	0.843459	-1.24317	0.775711
Ccl17	chemokine (C-C motif) ligand 17	0.00713	0	1.04	0.04	1.74	-0.83812	0.398255	-0.79057	1.230429
1700017B05Rik	RIKEN cDNA 1700017B05 gene	0.03	4.38	5.64	3.23	5.95	-0.33665	0.673301	-1.25843	0.921781
Fzd4	frizzled homolog 4 (Drosophila)	0.01	0.04	1.1	0.06	1.88	-0.81993	0.370654	-0.79747	1.246745
Elf2	E74-like factor 2	0.03	4.18	4.58	3.76	4.72	-0.30078	0.624705	-1.27255	0.948627
Tsc22d3	TSC22 domain family, member 3	0.01	3.89	7.22	2.02	6.15	-0.39979	1.03172	-1.20367	0.571745
RP23-71J17.1	LOC67647<***>4930523C07Rik	0.03	0.59	1.23	0.83	1.48	-1.1108	0.495781	-0.50833	1.123352
Klf2	Kruppel-like factor 2 (lung)	0.01	3.75	5.49	4.51	5.46	-1.2585	0.822057	-0.34975	0.786186
	Cluster average		2.3169	3.6816	2.1731	3.6956	-0.7544	0.783913	-0.91619	0.886675
	Cluster SD		1.5834	1.6596	1.4963	1.4851	0.230007	0.227635	0.213177	0.216226

Cluster 8

Feature ID	Name	FDR, P	U	D	L	(L+D)	Uz	Dz	Lz	(L+D)z
Rrad	Ras-related associated with diabetes	0.0023	3.82	3.04	4.54	4.15	-0.10596	-1.33036	1.024262	0.412059
Ltb	lymphotoxin B	0.00477	3.75	3.18	4.05	3.47	0.368506	-1.15912	1.172518	-0.38191
Rilpl2	Rab interacting lysosomal protein-like 2	0.02	6.93	6.44	7.53	6.79	0.016505	-1.06181	1.336888	-0.29158
Gadd45a	growth arrest and DNA-damage-inducible 45 alpha	0.02	3.81	3.24	5.14	3.57	-0.15598	-0.83991	1.439839	-0.44395
Egr2	early growth response 2	0.02	2.11	0.52	5.98	4.03	-0.44395	-1.11622	1.192328	0.367846
Enc1	ectodermal-neural cortex 1	0.03	3.53	1.72	2.79	2.1	1.247492	-1.02181	0.319709	-0.54539
B4galt5	UDP-Gal:betaGlcNAc beta 1,4-galactosyltransferase, polypeptide 5	0.04	4.96	4.25	6.06	4.83	-0.08599	-1.02528	1.36925	-0.25797
Bcl3	B-cell leukemia/lymphoma 3	0.04	4.6	3.35	6.07	5.5	-0.23609	-1.29005	1.003371	0.522765
Lfng	LFNG O-fucosylpeptide 3-beta-N-acetylglucosaminyltransferase	0.05	6.25	5.13	5.89	4.87	1.110564	-0.62906	0.551399	-1.0329
Hk2	hexokinase 2	0.04	3.97	3.85	4.44	3.47	0.093782	-0.20632	1.26918	-1.15664
Plekho2	pleckstrin homology domain containing, family O member 2	0.06	7	6.44	7.45	7.1	0.00597	-1.33132	1.080576	0.244771
Ptafr	platelet-activating factor receptor	0.07	6.07	5.51	6.76	6.23	-0.1409	-1.22924	1.200085	0.170053
Atf3	activating transcription factor 3	0.07	6.78	5.48	8.06	7.5	-0.15706	-1.32382	0.991746	0.489142
Ier3	immediate early response 3	0.07	4.16	1.81	7.12	5.84	-0.2495	-1.27365	1.040489	0.482656
Nr1d1	nuclear receptor subfamily 1, group D, member 1	0.07	3.43	2.42	3.68	2.36	0.67205	-0.8116	1.03929	-0.89974
Arl4c	ADP-ribosylation factor-like 4C	0.06	5.75	4.73	5.37	4.61	1.17815	-0.71431	0.473115	-0.93695
Bhlhe40	basic helix-loop-helix family, member e40	0.08	2.19	1.5	2.92	2.16	-0.00431	-1.19357	1.253891	-0.05602
Neurl3	neuralized homolog 3 homolog (Drosophila)	0.1	6.72	6.33	7.25	6.88	-0.19671	-1.21961	1.193381	0.222939
P2ry13	purinergic receptor P2Y, G-protein coupled 13	0.1	1.08	0.39	1.25	1	0.400225	-1.44081	0.853814	0.186772
Sertad1	SERTA domain containing 1	0.1	4.35	3.37	4.81	4.77	0.037325	-1.4258	0.724096	0.664376
4930471M23Rik	RIKEN cDNA 4930471M23 gene	0.11	5.81	5.15	5.36	4.86	1.287903	-0.36261	0.162551	-1.08784
	Cluster average		4.6224	3.7071	5.3581	4.5757	0.221048	-1.04792	0.985323	-0.15845
	Cluster SD		1.6992	1.8721	1.768	1.7915	0.548161	0.339759	0.351474	0.600366

Cluster 9

Feature ID	Name	FDR, P	U	D	L	(L+D)	Uz	Dz	Lz	(L+D)z
Ccnj	cyclin J	0.0023	1.38	3	0.55	1.09	-0.11856	1.417948	-0.90578	-0.39361
Sox4 (13 29040787.		0.00486	0.63	1.67	0.49	0.64	-0.41668	1.488156	-0.6731	-0.39837

Ddit4	DNA-damage-inducible transcript 4	0.00713	3.05	7.61	2.58	3.58	-0.50077	1.476287	-0.70454	-0.27098
Dyrk3	dual-specificity tyrosine-(Y)-phosphorylation regulated kinase 3	0.00773	0.38	1.28	0.49	0.7	-0.82929	1.4154	-0.55494	-0.03118
Gpt2	glutamic pyruvate transaminase (alanine aminotransferase) 2	0.01	1.46	2.47	1.39	1.63	-0.55653	1.469045	-0.69692	-0.21559
Ppm1d	protein phosphatase 1D magnesium-dependent, delta isoform	0.02	2.12	3.19	1.78	2.4	-0.42008	1.360062	-0.98573	0.045751
Sik1	salt inducible kinase 1	0.02	3.52	5.8	3.18	4.87	-0.67682	1.199345	-0.9566	0.434068
Pik3ip1	phosphoinositide-3-kinase interacting protein 1	0.02	0.62	2.05	0.29	0.7	-0.37957	1.460378	-0.80417	-0.27664
Net1	neuroepithelial cell transforming gene 1	0.03	1.14	3.08	1.01	1.66	-0.61473	1.43261	-0.75192	-0.06596
Arl4d	ADP-ribosylation factor-like 4D	0.03	1.03	3.05	0.27	0.79	-0.20924	1.448295	-0.83287	-0.40618
Ccbl1	cysteine conjugate-beta lyase 1	0.03	1.57	2.37	1.37	2.05	-0.5945	1.166974	-1.03486	0.462386
Exosc8	exosome component 8	0.03	2.3	2.96	2	2.31	-0.22853	1.40209	-0.96973	-0.20383
Abhd15	abhydrolase domain containing 15	0.03	2.96	4.69	2.09	3.24	-0.26373	1.33718	-1.06882	-0.00463
Klrg2	killer cell lectin-like receptor subfamily G, member 2	0.04	0.3	1.13	0.19	0.53	-0.56597	1.411949	-0.82811	-0.01787
Ppp1r3b	protein phosphatase 1, regulatory (inhibitor) subunit 3B	0.04	1.8	2.5	1.15	1.86	-0.04985	1.218989	-1.22805	0.05891
D230025D16Rik	RIKEN cDNA D230025D16 gene	0.05	3.28	3.67	2.6	3.23	0.191979	1.072824	-1.34385	0.07905
Rnf169	ring finger protein 169	0.06	2.78	4.53	2.06	3.71	-0.4546	1.168974	-1.12259	0.408213
Zfp69	zinc finger protein 69	0.06	0.56	1.37	0.54	0.62	-0.53154	1.494568	-0.58157	-0.38146
Dclre1b	DNA cross-link repair 1B, PSO2 homolog (S. cerevisiae)	0.07	1.18	2.21	1.13	1.76	-0.75932	1.24607	-0.85667	0.369927
Gm11827	predicted gene 11827	0.07	0.32	1.36	0.24	0.93	-0.74005	1.220847	-0.89089	0.410092
Ascl2	achaete-scute complex homolog 2 (Drosophila)	0.05	1.35	1.9	0.86	1.57	-0.16039	1.099846	-1.28315	0.343702
Foxred2	FAD-dependent oxidoreductase domain containing 2	0.05	1.94	2.72	1.65	1.72	-0.13753	1.45169	-0.72839	-0.58577
Ankrd9	ankyrin repeat domain 9	0.08	1.52	3.01	1.04	1.9	-0.41417	1.361704	-0.98627	0.038736
Napepld	N-acyl phosphatidylethanolamine phospholipase D	0.09	1.02	2.06	0.75	1.31	-0.46902	1.371664	-0.94689	0.044247
Marveld1	MARVEL (membrane-associating) domain containing 1	0.07	3.82	4.5	3.33	3.94	-0.16126	1.25369	-1.18086	0.088435
Ankrd16	ankyrin repeat domain 16	0.1	2.18	3.29	1.61	2.35	-0.25445	1.336754	-1.07155	-0.01075
Osbpl11	oxysterol binding protein-like 11	0.11	4.89	5.52	4.44	4.72	-0.00546	1.371196	-0.98879	-0.37694
	Cluster average		1.8185	3.0737	1.4474	2.067	-0.38225	1.339057	-0.9251	-0.03171
	Cluster SD		1.1857	1.5382	1.0741	1.2701	0.253164	0.124826	0.20527	0.300066

Cluster 10

Feature ID	Name	FDR, P	U	D	L	(L+D)	Uz	Dz	Lz	(L+D)z
Zfp691	zinc finger protein 691	0.00492	1.45	1.23	0.69	0.43	1.060872	0.594089	-0.55165	-1.10331
Rit1	Ras-like without CAAX 1	0.00713	4.69	4.31	3.78	3.79	1.241463	0.379808	-0.82197	-0.7993
Angptl4	angiopoietin-like 4	0.01	4.07	3.12	2.17	1.79	1.255248	0.325435	-0.60438	-0.9763
Armc7	armadillo repeat containing 7	0.02	3.08	2.26	2.04	1.72	1.386352	-0.02583	-0.40471	-0.95581
Fam84b	family with sequence similarity 84, member B(esophageal carcinoma cell)	0.02	1.89	1.44	0.97	0.72	1.225924	0.357159	-0.55022	-1.03286
CT030194.2	ENSMUST00000075093	0.02	1.42	1.09	0.67	0.59	1.23527	0.381575	-0.70494	-0.9119
Phf23	PHD finger protein 23	0.03	4.41	3.92	3.95	3.44	1.211411	-0.02524	0.050475	-1.23665
1810026J23Rik	RIKEN cDNA 1810026J23 gene	0.03	3.51	3.29	2.81	2.61	1.09256	0.564289	-0.5883	-1.06855
Cd300lb	CD300 antigen like family member B	0.03	6.66	5.39	5.04	4.29	1.328961	0.045478	-0.30824	-1.0662
Pdp2	pyruvate dehyrogenase phosphatase catalytic subunit 2	0.03	1.7	1.36	1.01	0.71	1.177607	0.384763	-0.4314	-1.13097
Dynlt1c	dynein light chain Tctex-type 1C	0.03	1.88	1.63	1.18	1.06	1.153247	0.501695	-0.6711	-0.98384
Sema4b	sema domain, immunoglobulin domain (Ig), transmembrane domain (TM) ar	0.04	2.84	2.51	2.26	1.73	1.078873	0.373867	-0.16023	-1.29251
Tsku	tsukushin	0.04	4	3.29	3.35	2.78	1.289227	-0.12992	-0.00999	-1.14931
Snx33	sorting nexin 33	0.04	2.54	2.34	1.86	1.57	1.04497	0.593091	-0.49142	-1.14664
Pdp1	pyruvate dehyrogenase phosphatase catalytic subunit 1	0.05	2.87	2.39	2.06	1.67	1.223591	0.280099	-0.36855	-1.13514
Med22	mediator complex subunit 22	0.05	4.4	4.11	3.7	3.49	1.163857	0.453292	-0.5513	-1.06585
Sertad3	SERTA domain containing 3	0.04	3.23	2.1	1.72	1.3	1.378517	0.015082	-0.44342	-0.95018
B430306N03Rik	RIKEN cDNA B430306N03 gene	0.06	4.02	3.55	3.19	2.9	1.25238	0.279457	-0.46576	-1.06608
Spsb2	splA/ryanodine receptor domain and SOCS box containing 2	0.06	4.18	3.64	3.25	2.98	1.281228	0.244729	-0.50385	-1.0221
Ovca2	candidate tumor suppressor in ovarian cancer 2	0.07	1.24	0.83	0.36	0.19	1.232285	0.368632	-0.62141	-0.97951
Smcr7l	Smith-Magenis syndrome chromosome region, candidate 7-like (human)	0.06	2.87	2.57	2.24	1.91	1.139879	0.416146	-0.37996	-1.17607
Mlst8	MTOR associated protein, LST8 homolog (S. cerevisiae)	0.07	3.15	2.84	2.28	2.07	1.135693	0.512569	-0.61307	-1.03519
Chst14	carbohydrate (N-acetylgalactosamine 4-0) sulfotransferase 14	0.07	4.12	3.78	3.31	3.29	1.236856	0.387298	-0.78709	-0.83706
Iffo2	intermediate filament family orphan 2	0.08	2.65	2.1	1.87	1.58	1.324317	0.11036	-0.3973	-1.03738
Slc35c1	solute carrier family 35, member C1	0.07	3.91	3.71	3.35	3.02	1.047667	0.539707	-0.37462	-1.21275
Slc30a1	solute carrier family 30 (zinc transporter), member 1	0.09	3.97	3.42	3.29	2.92	1.310201	0.045972	-0.25285	-1.10333
D17H6S53E	DNA segment, Chr 17, human D6S53E	0.09	3.51	3.28	2.69	2.34	1.03532	0.606269	-0.49434	-1.14725
Gpr157	G protein-coupled receptor 157	0.1	2.75	2.46	2.05	1.91	1.191262	0.436145	-0.63143	-0.99597
Engase	endo-beta-N-acetylglucosaminidase	0.1	4.32	3.84	3.14	2.97	1.199708	0.434446	-0.68156	-0.95259
Atmin	ATM interactor	0.09	3.62	3.38	2.95	2.7	1.103478	0.524604	-0.51254	-1.11554
Usf1	upstream transcription factor 1	0.1	5.01	4.7	4.11	3.95	1.141506	0.517952	-0.66881	-0.99065
Ss18l1	synovial sarcoma translocation gene on chromosome 18-like 1	0.09	1.84	1.56	1	0.83	1.126895	0.534349	-0.65074	-1.0105
Mapk7	mitogen-activated protein kinase 7	0.09	3.74	3.26	3.16	2.86	1.327729	0.013688	-0.26007	-1.08135
Lhfpl2	lipoma HMGIC fusion partner-like 2	0.09	4.44	3.79	3.71	3.31	1.340605	-0.04807	-0.21898	-1.07355
Zfp710	zinc finger protein 710	0.1	5.36	4.87	4.88	4.27	1.153789	0.056009	0.078413	-1.28821
	Cluster average		3.4097	2.9531	2.574	2.2769	1.203678	0.315685	-0.4585	-1.06087
	Cluster SD		1.2231	1.1363	1.1903	1.1347	0.097842	0.22022	0.220727	0.111613

Cluster 11

Feature ID	Name	FDR, P	U	D	L	(L+D)	Uz	Dz	Lz	(L+D)z
2410075B13Rik	RIKEN cDNA 2410075B13 gene	0.00492	1.18	1.4	0.3	0.24	0.670904	1.039902	-0.80509	-0.90572
Med18	mediator of RNA polymerase II transcription, subunit 18 homolog (yeast)	0.00661	1.55	1.69	0.48	0.6	0.748329	0.971236	-0.95531	-0.76425
Zfp212	Zinc finger protein 212	0.00669	2.37	2.42	1.58	1.69	0.803985	0.917223	-0.98517	-0.73604
2810008D09Rik	RIKEN cDNA 2810008D09 gene	0.01	3.24	3.32	1.39	1.74	0.816728	0.896652	-1.03152	-0.68186
1110054O05Rik	RIKEN cDNA 1110054O05 gene	0.01	2.28	2.38	1.22	1.46	0.765461	0.937475	-1.05788	-0.64505
Frat2	frequently rearranged in advanced T-cell lymphomas 2	0.01	2.13	2.29	1.2	1.31	0.713595	1.000829	-0.95595	-0.75848
Mett11d1		0.01	1.84	1.86	0.94	1.04	0.842927	0.883067	-0.96335	-0.76265
Smug1	single-strand selective monofunctional uracil DNA glycosylase	0.02	1.66	1.74	0.85	0.87	0.781666	0.946227	-0.88452	-0.84338
Zfp161	zinc finger protein 161	0.02	1.74	1.78	0.8	0.86	0.827534	0.901919	-0.92052	-0.80894
Zfp324	zinc finger protein 324	0.02	1.2	1.25	0.39	0.44	0.811023	0.917737	-0.91774	-0.81102
Zkscan14	zinc finger with KRAB and SCAN domains 14	0.03	2.36	2.41	0.7	0.89	0.835608	0.889869	-0.96583	-0.75964
Txnip	thioredoxin interacting protein	0.03	7.29	7.76	4.04	4.72	0.724202	0.978688	-1.03554	-0.66735

Lysmd4	LysM, putative peptidoglycan-binding, domain containing 4	0.03	1.72	1.89	0.75	0.9	0.706407	1.002924	-0.98548	-0.72385
Zfp764	zinc finger protein 764	0.03	1.95	2.06	1.24	1.3	0.731123	0.988479	-0.92999	-0.78961
Triap1	TP53 regulated inhibitor of apoptosis 1	0.03	5.06	4.93	4.08	4.37	0.971701	0.690987	-1.14445	-0.51824
AC099934.2		0.03	1.46	1.39	0.66	0.83	0.938184	0.763057	-1.06328	-0.63797
5730403M16Rik		0.03	2.54	2.52	1.73	1.83	0.885038	0.839062	-0.97699	-0.74711
Zfp553	zinc finger protein 553	0.04	1.83	1.98	0.78	0.81	0.745464	0.978421	-0.88524	-0.83865
Commd5	COMM domain containing 5	0.04	3.71	3.62	2.78	3.06	0.934903	0.733367	-1.14764	-0.52063
Lipt2	lipoyl(octanoyl) transferase 2 (putative)	0.05	1.37	1.57	0.44	0.7	0.652262	1.024983	-1.08089	-0.59635
Etaa1	Ewing's tumor-associated antigen 1	0.05	1.55	1.65	0.7	0.78	0.761117	0.961411	-0.94138	-0.78115
Zfp866	zinc finger protein 866	0.05	1.82	2.02	0.98	1.24	0.626992	1.038134	-1.09981	-0.56532
Uck1	uridine-cytidine kinase 1	0.05	3.67	3.65	2.81	2.95	0.88102	0.836969	-1.01317	-0.70482
Taf8	TAF8 RNA polymerase II, TATA box binding protein (TBP)-associated factorq	0.05	3.46	3.6	2.66	2.96	0.663446	0.98373	-1.16675	-0.48043
Pelo	pelota homolog (Drosophila)	0.07	2.5	2.64	1.52	1.74	0.723259	0.9764	-1.04873	-0.65093
Mlycd	malonyl-CoA decarboxylase	0.06	2.92	3.02	2.19	2.29	0.740476	0.975548	-0.97555	-0.74048
Zfp58	zinc finger protein 58	0.06	1.62	1.69	0.76	0.9	0.785507	0.931164	-1.00399	-0.71268
Eid2b	EP300 interacting inhibitor of differentiation 2B	0.05	1.45	1.55	0.71	0.8	0.743741	0.974359	-0.96283	-0.75527
Eid2	EP300 interacting inhibitor of differentiation 2	0.07	2.02	2.18	1.37	1.55	0.627679	1.046132	-1.07228	-0.60153
Gm16009	predicted gene 16009	0.07	3.82	3.84	2.79	3.06	0.828551	0.866	-1.10005	-0.5945
Rpusd2	RNA pseudouridylate synthase domain containing 2	0.06	1.21	1.36	0.44	0.41	0.709385	1.009126	-0.82928	-0.88923
Rbak	RB-associated KRAB repressor	0.05	1.71	1.62	0.55	0.85	0.922803	0.765358	-1.10649	-0.58167
Cdkn2aipnl	CDKN2A interacting protein N-terminal like	0.06	2.35	2.52	1.63	1.95	0.592717	1.016977	-1.20415	-0.40554
Cnr2	cannabinoid receptor 2 (macrophage)	0.06	2.76	3.46	1.56	2	0.375541	1.210078	-1.05509	-0.53053
Nr2c2ap	nuclear receptor 2C2-associated protein	0.06	3.27	3.16	2.05	2.52	0.909161	0.716839	-1.22387	-0.40213
Zfp472	zinc finger protein 472	0.06	1.76	2.56	0.37	1.03	0.349839	1.197932	-1.12372	-0.42405
2310044G17Rik	RIKEN cDNA 2310044G17 gene	0.09	3.92	4.04	3.23	3.44	0.681359	0.992837	-1.10964	-0.56455
Itpripl1	inositol 1,4,5-triphosphate receptor interacting protein-like 1	0.08	3.5	3.91	2.23	2.56	0.572332	1.09379	-1.04292	-0.62321
Zbtb48	zinc finger and BTB domain containing 48	0.08	1.75	1.74	0.79	0.93	0.870355	0.850906	-0.99678	-0.72449
E130102H24Rik	RIKEN cDNA E130102H24 gene (mir101a)	0.07	1.79	2.09	0.42	0.94	0.625295	1.016105	-1.1594	-0.482
Txnl4b	thioredoxin-like 4B	0.08	2.72	2.87	1.97	2.26	0.639158	1.000945	-1.16978	-0.47032
Xrcc6bp1	XRCC6 binding protein 1	0.07	2.03	2.38	1.09	1.38	0.525449	1.118698	-1.06785	-0.5763
Fbxo32	F-box protein 32	0.08	1.55	2.02	0.67	0.83	0.447704	1.192555	-0.94691	-0.69335
Zfp251	zinc finger protein 251	0.07	1.73	2.02	1.24	1.2	0.460161	1.191376	-0.77534	-0.8762
Card6	caspase recruitment domain family, member 6	0.09	1.2	1.7	0.54	0.68	0.321193	1.26588	-0.92579	-0.66128
Zfp746	zinc finger protein 746	0.08	3.43	3.83	2.51	3.02	0.411376	1.119119	-1.21643	-0.31406
Patz1	POZ (BTB) and AT hook containing zinc finger 1	0.09	2.2	2.38	1.41	1.45	0.677028	1.035454	-0.89607	-0.81642
Zfp652	zinc finger protein 652	0.09	3.4	3.73	2.93	2.99	0.366493	1.246076	-0.88625	-0.72632
Arl11	ADP-ribosylation factor-like 11	0.1	6.11	6.17	5.27	5.54	0.768585	0.905223	-1.14434	-0.52947
Atg14	VATG14 autophagy related 14 homolog (S. cerevisiae)	0.11	2.89	2.95	1.98	2.04	0.807172	0.921126	-0.92113	-0.80717
Sap25	sin3 associated polypeptide	0.11	5.23	5.15	4.33	4.53	0.938523	0.759757	-1.0726	-0.62568
	Cluster average		2.5455	2.7012	1.5696	1.7741	0.71099	0.970943	-1.01864	-0.66329
	Cluster SD		1.2848	1.2837	1.1577	1.2173	0.164521	0.133437	0.107425	0.138736

Cluster 12

Feature ID	Name	FDR, P	U	D	L	(L+D)	Uz	Dz	Lz	(L+D)z
Tmem81	transmembrane protein 81	0.00423	2.54	2.5	1.54	1.51	0.900272	0.830686	-0.83938	-0.89157
Zfp623	zinc finger protein 623	0.00492	1.8	1.75	0.69	0.68	0.905256	0.825848	-0.85761	-0.87349
Lcmt2	leucine carboxyl methyltransferase 2	0.00661	2.26	2.25	1.16	1.13	0.87365	0.858049	-0.84245	-0.88925
Gtf3c4	general transcription factor IIIC, polypeptide 4	0.00729	2.46	2.33	1.6	1.47	0.985738	0.726857	-0.72686	-0.98574
Zscan2	zinc finger and SCAN domain containing 2	0.00918	1.18	1.22	0.34	0.35	0.82503	0.906015	-0.87565	-0.8554
Zfp12	zinc finger protein 12	0.00954	1.77	1.74	0.71	0.69	0.890392	0.841154	-0.84936	-0.88219
Bat4		0.01	3.1	2.94	2.04	2.22	1.00351	0.697679	-1.02262	-0.67856
Rab19	RAB19, member RAS oncogene family	0.01	1.64	1.63	1.01	0.75	0.852547	0.830259	-0.55165	-1.13116
Tmem177	transmembrane protein 177	0.01	1.66	1.49	0.91	0.83	1.056263	0.645829	-0.75447	-0.94762
Kbtbd4		0.01	3.25	3.06	2.49	2.43	1.080796	0.616726	-0.77549	-0.92204
Mrm1	mitochondrial rRNA methyltransferase 1 homolog (S. cerevisiae)	0.02	2.51	2.42	1.34	1.24	0.929318	0.797083	-0.78974	-0.93666
Trmt12	tRNA methyltranferase 12 homolog (S. cerevisiae)	0.02	1.78	1.53	0.69	0.74	1.076728	0.624321	-0.89577	-0.80528
Zscan22	zinc finger and SCAN domain containing 22	0.02	1.69	1.52	0.7	0.63	1.013005	0.702715	-0.79398	-0.92174
Nhlrc1	NHL repeat containing 1	0.02	1.28	1.23	0.35	0.37	0.91358	0.816905	-0.88458	-0.84591
5730409E04Rik	RIKEN cDNA 5730409E04Rik gene	0.02	3.17	3.2	2.28	2.28	0.837087	0.894488	-0.86579	-0.86579
Zfp790	zinc finger protein 790	0.02	1.63	1.52	0.79	0.76	0.980139	0.743182	-0.82935	-0.89397
Zfp688	zinc finger protein 688	0.03	2.22	2.12	1.07	1.04	0.941634	0.786633	-0.84088	-0.88738
Tmem186	transmembrane protein 186	0.03	2.39	2.27	1.28	1.48	0.961043	0.745482	-1.0329	-0.67363
Brf2	BRF2, subunit of RNA polymerase III transcription initiation factor, BRF1-like	0.03	2.9	2.79	1.82	1.83	0.95662	0.770375	-0.87196	-0.85503
2900076A07Rik	RIKEN cDNA 2900076A07 gene, mir1839	0.03	1.45	1.33	0.53	0.44	0.97419	0.746087	-0.7746	-0.94568
Bet1l	blocked early in transport 1 homolog (S. cerevisiae)-like	0.03	3.91	3.76	2.93	3.09	1.005868	0.69637	-1.01618	-0.68605
2310046O06Rik	RIKEN cDNA 2310046O06 gene	0.03	2.88	2.89	1.97	1.94	0.856466	0.875085	-0.83785	-0.8937
Asb8	ankyrin repeat and SOCS box-containing 8	0.03	3.78	3.66	2.86	2.83	0.979911	0.743551	-0.83219	-0.89128
Zfp780b	zinc finger protein 780B	0.03	1.35	1.17	0.4	0.56	1.041348	0.650842	-1.01965	-0.67254
Surf6	surfeit gene 6	0.03	2.9	2.76	2.13	1.96	1.00097	0.697974	-0.66551	-1.03343
Giyd2		0.03	2.02	1.96	1.27	1.13	0.923259	0.792916	-0.70602	-1.01015
9930012K11Rik	RIKEN cDNA 9930012K11 gene	0.03	1.1	0.88	0.24	0.34	1.105735	0.576905	-0.96151	-0.72113
Zfp61	zinc finger protein 61	0.03	1.33	1.32	0.47	0.47	0.876124	0.855867	-0.866	-0.866
Pygo2	pygopus 2	0.03	3.59	3.52	2.86	2.73	0.936991	0.778944	-0.71121	-1.00473
Clk2	CDC-like kinase 2	0.03	3.01	2.89	2.2	2.3	1.002089	0.708794	-0.97765	-0.73324
D330012F22Rik	RIKEN cDNA D330012F22 gene	0.04	1.71	1.63	0.72	0.67	0.934898	0.793113	-0.8197	-0.90831
Zfp707	zinc finger protein 707	0.04	1.47	1.46	0.43	0.45	0.87437	0.857474	-0.88282	-0.84903
Alkbh4	alkB, alkylation repair homolog 4 (E. coli)	0.04	2.19	1.97	1.33	1.16	1.065077	0.620874	-0.67135	-1.0146
Tcta	T-cell leukemia translocation altered gene	0.04	3.48	3.33	2.48	2.55	1.003345	0.713919	-0.92616	-0.7911
Fam58b	family with sequence similarity 58, member B	0.04	4	3.87	3.02	3.07	0.986502	0.73504	-0.90913	-0.81241
Fn3krp	fructosamine 3 kinase related protein	0.04	1.88	2.07	1.44	1.15	0.587061	1.042334	-0.46725	-1.16214
Klhdc5	kelch domain containing 5	0.04	1.44	1.39	0.63	0.54	0.914684	0.810742	-0.76917	-0.95626
Wdr85	WD repeat domain 85	0.04	2.3	2.09	1.52	1.44	1.093699	0.5971	-0.75081	-0.93999

Tcfap4	transcription factor AP4	0.05	2.12	1.92	0.94	0.85	1.01043	0.705395	-0.78928	-0.92655
Zfp84	zinc finger protein 84, Mir1964	0.05	2.53	2.23	1.36	1.33	1.094079	0.602358	-0.82363	-0.8728
Cbx8	chromobox homolog 8 (Drosophila Pc class)	0.05	1.31	1.08	0.34	0.24	1.065849	0.633875	-0.75595	-0.94377
Mad2l1bp	MAD2L1 binding protein	0.05	3.44	3.28	2.38	2.42	1.00299	0.716421	-0.89553	-0.82388
Pars2	prolyl-tRNA synthetase (mitochondrial)(putative)	0.05	1.55	1.55	0.58	0.54	0.865672	0.865672	-0.8307	-0.90065
Zswim3	zinc finger, SWIM domain containing 3	0.05	1.75	1.49	0.88	0.79	1.119008	0.562181	-0.74422	-0.93697
Zfp28	zinc finger protein 28	0.05	2.06	1.97	1.2	1.26	0.960765	0.763122	-0.92782	-0.79606
Zfp768	zinc finger protein 768	0.06	2.97	2.84	2.17	2.13	1.007005	0.711162	-0.81357	-0.9046
AW209491	expressed sequence AW209491	0.06	2.07	2.1	1.3	1.29	0.832805	0.898553	-0.85472	-0.87664
Acrbp	proacrosin binding protein	0.06	1.29	1.05	0.47	0.53	1.138093	0.53778	-0.91298	-0.7629
Casp2	caspase 2	0.07	2.58	2.63	1.69	1.61	0.818689	0.909152	-0.79155	-0.93629
Fam78a	family with sequence similarity 78, member A	0.08	2.72	2.59	1.68	1.56	0.967758	0.751778	-0.76008	-0.95945
Thap11	THAP domain containing 11	0.08	4.04	3.92	3.12	3	0.970762	0.74674	-0.74674	-0.97076
Gm10521	predicted gene 10521	0.08	2.37	2.4	1.75	1.52	0.812191	0.879873	-0.58658	-1.10548
2410131K14Rik	RIKEN cDNA 2410131K14 gene	0.08	1.84	1.75	1.08	0.98	0.960512	0.758299	-0.74707	-0.97175
B3galt4	UDP-Gal:betaGlcNAc beta 1,3-galactosyltransferase, polypeptide 4	0.09	3.08	2.97	1.91	1.75	0.939562	0.781169	-0.74517	-0.97556
Tab1	TGF-beta activated kinase 1/MAP3K7 binding protein 1	0.09	3.61	3.44	2.66	2.52	1.009578	0.698939	-0.72635	-0.98217
Rnf144b	ring finger protein 144B	0.09	4.38	4.36	3.34	3.44	0.881317	0.846064	-0.95182	-0.77556
Kctd21	potassium channel tetramerisation domain containing 21	0.09	2.66	2.55	1.76	1.69	0.968975	0.753647	-0.7928	-0.92982
Irf2bp1	interferon regulatory factor 2 binding protein 1	0.09	3.21	3.27	2.47	2.31	0.796883	0.917928	-0.69601	-1.0188
Mcat	malonyl CoA:ACP acyltransferase (mitochondrial)	0.09	3.08	2.84	2.27	2.17	1.113062	0.567889	-0.7269	-0.95405
Zfp646	zinc finger protein 646	0.09	3	2.9	2.26	2.11	0.965913	0.74258	-0.68675	-1.02175
2410002I01Rik	RIKEN cDNA 2410002I01 gene	0.09	2.98	2.9	2.14	2.15	0.950731	0.776883	-0.87467	-0.85294
Ftsj2	FtsJ homolog 2 (E. coli)	0.1	2.73	2.43	1.58	1.37	1.072103	0.614265	-0.68294	-1.00343
Cd3eap	CD3E antigen, epsilon polypeptide associated protein	0.1	2.51	2.62	1.78	1.6	0.746386	0.961033	-0.67809	-1.02933
Nup62	nucleoporin 62	0.1	2.17	1.9	1.17	1.26	1.118003	0.56413	-0.93338	-0.74875
Iffo1	intermediate filament family orphan 1	0.1	3.1	2.86	2.21	2.24	1.113117	0.576136	-0.87819	-0.81106
Zbtb12	zinc finger and BTB domain containing 12	0.1	2.59	2.35	1.76	1.78	1.129991	0.552974	-0.86552	-0.81744
Zfp282	zinc finger protein 282	0.1	1.79	1.74	1.02	0.98	0.921017	0.808008	-0.81931	-0.90972
Gm11641	predicted gene 11641	0.11	2.9	2.94	2.05	1.96	0.825778	0.901278	-0.77859	-0.94846
Mtif3	mitochondrial translational initiation factor 3	0.11	1.57	1.46	0.69	0.76	0.97996	0.740415	-0.93641	-0.78397
	Cluster average		2.4206	2.3158	1.5109	1.4694	0.96093	0.750723	-0.81476	-0.89689
	Cluster SD		0.8057	0.8075	0.8036	0.8047	0.102843	0.110419	0.108847	0.104008

Table S3 qPCR primers used in this study

Expression primers

t64_mKdm6b_RT_F<br>t65_mKdm6b_RT_R	CTCTAAGCATGGAGGTGGTTCTC<br>TTCACTTCGGCTCAGTGAGTTTG	
s42_mMaff_F1<br>s43_mMaff_R1	CTGTCGGATGAAGCGCTGATGGG<br>CGTAGCCGCGGTTCTTGAGTGTG	
o93_mDusp1_1_F<br>o94_mDusp1_1_R	TTTGTGTAGGTCGGTGGTCTGCCTT<br>TGGCTTTGTCTGTCAGTGCCGAAAG	
p20_mCited2_1_F<br>p21_mCited2_1_R	GCTGCCGCCCAATGTCATAGACA<br>GAGTTCGGGCAGCTCCTTGATGC	
m89_mMt2_F<br>m90_mMt2_R	GGCGCCTGCAAATGCAAACAATGC<br>CACTTGTCGGAAGCCTCTTTGCAG	
u55_mEdn1_F1<br>u56_mEdn1_R1	AACCGAGCACATTGACTACAGAG<br>TAGAAGCCACACAGATGGTCTTG	
v94_mIRF1_F<br>v95_mIRF1_R	CCCAAGACATGGAAGGCAAACTT<br>GCTGCCCTTGTTCCTACTCTGAT	
q25_mRasgef1b_R<br>q26_mRasgef1b_F	AGGTCCAGCCTTTTGGGCAGAGT<br>GCCAGAAAACAAAGGCCAGCCC	
k47_mIL1b_F<br>k48_mIL1b_R	GGGCTGCTTCCAAACCTTTGACC<br>GTAGCTGCCACAGCTTCTCCACAGCC	
q63_mHspa1a_F<br>q64_mHspa1a_R	ACTTTGTGTATTGCACGTGGGCTTT<br>GCGGGGCAGTGCTGAATTGAAGA	
m77_mIL12-p40_F<br>m78_mIL12-p40_R	AGGACTCCCCATTCCTACTTCTCC<br>CACCCCTCCTCTGTCTCCTTCAT	
u85_mEtv3_F1<br>u86_mEtv3_R1	CTCCTGTTCCCTCTCGGATTAAG<br>CTTTCCCCTCTTCTATGGTCCTG	
p48_mAtf3_F<br>p49_mAtf3_R	TGGGACCACATCCTCCACTGTGG<br>ACCCTGGCCATTGGACAACCTCA	
r98_mGadd45a_F<br>r99_mGadd45a_R	GCAGTTGAACTCGGCCCCTTGAC<br>ATCTCCCGGAACGGTGATGGCA	
s11_mAngptl4_F<br>s12_mAngptl4_R	GGGGCAGGGCATGGATACACACT<br>TGGGACCAAGACCATGACCTCCG	
s14_mSnx33_F<br>s15_mSnx33_R	GGCTCTCCCATATTCCCAGGGACC<br>GCCCAAGCACGACCTCTTCCAAA	
r2_mTxnip_F<br>r3_mTxnip_R	GTGGCCCGACACACTTACCTTGC<br>TCTGCACTCTGAGGCTCTTGCCA	
s16_mFrat2_F<br>s17_mFrat2_R	GAGTGACTGAGTCCGGTCCGCTT<br>CCGGAAACCTCATCAAGGAGGCG	
u51_mCbx8_F<br>u52_mCbx8_R	AAGAATCCTTGGTCCGTGTTGC<br>TGACTAACCTGGAGAAAGTGGTC	
v60_mZfp623_F<br>v61_mZfp623_R	CCTCTGAGTCTCGGGAATTTCAT<br>TCCTGTTTGAATCTTCCAGTGGG	
o87_mDdit4_F<br>o88_mDdit4_R	GCGCCTTCATTCGGACCTTA<br>AAGAGGAGGACGAGAAACGA	
o99_mSik1_F<br>p1_mSik1_R	GCTCTCCACTTCTGAAACGCGCA<br>CATGCCGTTTGTCTTCCCCTGGG	
p46_mNfil3_F<br>p47_mNfil3_R	CGGTGGAGCACACTCAGGAAAGC<br>CAAAGCTCTCCAACTCCACGGGC	
w15_mNcoa5_F<br>w16_mNcoa5_R	TCTCTGCAGCTTCACGACAATTA<br>TAGATTGGTGGCAGTTCTTACCC	

o89_mKLF4_F<br>o90_mKLF4_R	CCTTGGTGTTAGCTGAGATTG<br>GAACAGGGAGGGAGAGAGAAA	
u15_mKLF9_F2<br>u16_mKLF9_R2	CCCACTGTGTGAGAAGAGATTCAT<br>TCTTGATCATGCTGGGATGGAAC	
L71_mGILZ_F2_397<br>L72_mGILZ_R2_564	TGTACGCTGTGAGAGAGGAGG<br>CTTTCAGGTGCTGGCTCTTC	
p54_mFKBP5_F<br>p55_mFKBP5_R	TGGGATCGACAAAGCCCTGGTGA<br>GCTCAGCATTGGGGTCAATGCCA	
p10_mKlf2_F<br>p11_mKlf2_R	ACCCTTTCAGTGCCACTTGTGCG<br>CCGAGTCGACCCAGGCTACATGT	
v88_mTiparp_F<br>v89_mTiparp_R	CCCTTGAGCTTGTGTTTGAACTT<br>ATAGGGCAATACGGTGTGATGTT	
p66_mTGFb3_F<br>p67_mTGFb3_R	CTTTGCGGAGGACGGAGTAACGC<br>ACTTGGACCCACAGTCACCAGCA	
p64_mIL15ra_F<br>p65_mIL15ra_R	CCACGTGTCCACCTCCCGTATCT<br>CAGGGTGGATGTTCCAGCTTTCCG	
v78_mJunB_F<br>v79_mJunB_R	AGGTGGGTTTCAGGAGTTTGTAG<br>CACGAAAATGGAACAGCCTTTCT	
v84_mBCL3_F<br>v85_mBCL3_R	TGTCCTGTGTTCTGCTGACCTTA<br>CCGGAGGCCCTTTACTACCA	
p48_mATF3_F<br>p49_mATF3_R	TGGGACCACATCCTCCACTGTGG<br>ACCCTGGCCATTGGACAACCTCA	
v70_mPPARg1_F<br>v71_mPPARg1_R	AGAAGCGGTGAACCACTGATATTC<br>AGAGGTCCACAGAGCTGATTCC	Lefterova at al. Mol Cell Biol.<br>(2010) 30, 2078-89
r92_mMir22_hg_F<br>r93_mMir22_hg_R	TCCCTCGACCTCGCTTGCTTTCT<br>GCTTCCAGGCTGCCCTTTCTCAC	
q65_mMir155_hg_F<br>q66_mMir155_hg_R	GGAGGCTTGCTGAAGGCTGTATGC<br>CATCCTCCCACGGTGGCCATTTG	
s9_mMir101a_hg_F<br>s10_mMir101a_hg_R	CTGTGATAACTGAGCCAGGGCAGC<br>CAGTCCTTCACTCCGAGGGGCAC	
s7_mZfp84_F<br>s8_mZfp84_R	GCCCTGGCACCCCTTGAAGAAAG<br>ACCACCAACTCCTGAAATGAACGCA	
s5_mMir1839_hg_F<br>s6_mMir1839_hg_R	TGGGTACTCTCCTTGCTGATGGCA<br>AGCCATGAACGCTGAAGACATGC	
r90_mIfrd1-mir1938_F<br>r91_mIfrd1-mir1938_R	CCTCCGCAAAACTGGAAGGATCGC<br>CCTAATAGGTGGCCCGCATCGGA	
u59_Mir125a_hg_F<br>u60_Mir125a_hg_R	GTCCCTGAGACCCTTTAACCTG<br>CAGGCTCCCAAGAACCTCAC	
u57_mIcam5_F1<br>u58_mIcam5_R1	GCTCAACTGCAGCACTAACTG<br>CTCAGGCTCTCGGATGTCCAC	
v50_mZkscan14_F<br>v51_mZkscan14_R	GTGTTCACAGTTCGTGTTTCCAA<br>GGCACTTCCTTTACCAGCATTTT	
v54_mZfp472_F<br>v55_mZfp472_F	AAACTCACCAACAGAGTCCAGAG<br>CCAGTTTGACCTTTGAAGTGCAT	
v56_mRbak_F<br>v57_mRbak_R	TCACTGTCTAAGGCTTCCATGC<br>GGGTAACTCCTCTCAACACGAT	
v58_mZfp688_F<br>v59_mZfp688_R	TGTTCAACCACCTCTTTAGGTTGC<br>ACATTCCAAATGAGACAGAGTGG	
v60_mZfp623_F<br>v61_mZfp623_R	CCTCTGAGTCTCGGGAATTTCAT<br>TCCTGTTTGAATCTTCCAGTGGG	

v62_mZfp746_F<br>v63_mZfp746_R	AAAGACAGAGAGTAGGGCAGAGA<br>GCGCATTTTAGAACGGTAGTGAG	
v64_mZfp12_F<br>v65_mZfp12_R	TTTAGTGGGGATGGAGATGAGGA<br>AGGATTTCTGGCACTCAACGTAT	
v66_mZfp553_F<br>v67_mZfp553_R	TCAAGGAAGAGGAACAGTTAGCC<br>TCTCTGCGGTCCCATAAATCATC	
v68_mZfp707_F<br>v69_mZfp707_R	TGACTGCCTAAGTAGATTGGCT<br>AGAACTGGCCCAGATACAAATCA	
v86_mHprt_F<br>v87_mHprt_R	TGCTCGAGATGTCATGAAGGAGA<br>AATGTAATCCAGCAGGTCAGCA	
v2_mJdp2_F<br>v3_mJdp2_R	AATACGCTGACATCCGCAACATT<br>GCTTAGAACCAAACCTGGAAACG	
p52_mPer1_1F<br>p53_mPer1_1R	TGGACTCTGATATCCAGGAGCTCTC<br>GGGGACATCAGAGGGCCAACTCCA	
m89_mMt2_F<br>m90_mMt2_R	GGCGCCTGCAAATGCAAACAATGC<br>CACTTGTCGGAAGCCTCTTTGCAG	
v80_mTGIF1_F<br>v81_mTGIF1_R	CCAACTACGCAGGAATGAAATGG<br>CTGAGAAAGGATGGCAAAGATCC	
b90_mActb_F<br>b91_mActb_R	AGGTGTGCACTTTTATTGGTCTCAA<br>TGTATGAAGGCTTTGGTCTCCCT	

ChIP primers

p82_mPer1_p1145_F<br>p83_mPer1-p1145_R	TACAGGACCGCTGTCGTTGGGTT<br>CGTGTTCTCTTGGCTGATGGCCC	chr11: 68910509-68910585
p72_mCited_p588_F<br>p73_mCited_p588_R	CCAAAGCCCGGAGAAACACAGCT<br>GGAGCAAATTCCGAGCCGTTCCC	chr10:17443015-17443120
w3_mKlf2_p7960wa_F<br>w4_mKlf2_p7960wa_R	AGATCTTGCCTTGGCATTCAAAC<br>CAGAAGATTTTCCAGCGTCATCC	chr8:74827635-74827739
u81_mKLF9_4126wa_F<br>u82_mKLF9_4126wa_R	GCTCGTTGGACAAAGAGATGATG<br>CTGTGGTTGTTGTGGAACAGTTT	chr19:23209504+23209609
u75_mNfil3_2021wa_F<br>u76_mNfil3_2021wa_R	TTCCAAATGACTCAGGGATGTGT<br>CCAACACCTACATCTCCTGTTGA	chr13:53080928-53081070
u77_mJdp2_1702wa_F<br>u78_mJdp2_1702wa_R	AAGAACAGGACGTTCTTGTAGCA<br>GCATCTGTCACTAACAGGCTTTC	chr12:86873677-86873806
w9_mTiparp_p5251wa_F<br>w10_mTiparp_p5251wa_R	GGAGGCAGATAAACAAAGGCAAC<br>AGATGCAGGGTCACCGTGTT	chr3:65331894-65331999
w13_mNcoa5_p5035wa_F<br>w14_mNcoa5_p5035wa_R	CTTACAAAGTGCTCATTCTGTCCC<br>CCAGCTGAAGAGTTGTAAAAGGAC	chr2:164852840-164852929
u89_mKLF4+550_F<br>u90_mKLF4+550_R	TAATCTTCGTTGACTTTGGGGCT<br>CAAGTCCAACATCTCACCTACCT	chr4:55544718-55544821
u91_mKLF4+2.9_F<br>u92_mKLF4+2.9_R	CAGAAGCCTTTTGCTTGATGGAT<br>AACTCCCTATCTTTTAAAGGCCCA	chr4:55542357-55542435
u93_mKLF4+4.1_F<br>u94_mKLF4+4.1_R	CATGCAGTAGAATTTGCTCTCTGG<br>TGAAGCCATTGCTAGAAGGAAGAA	chr4:55541199-55541286
u95_mKLF4_GRE3_F<br>u96_mKLF4_GRE3_R	CAAACGTGTGTGTATATGTACATGTTACATTTT<br>CTTCAGAGACATTCAGGAAGCTTTGAG	chr4:55539368-55539632
u97_mKLF4_GRE2_F<br>u98_mKLF4_GRE2_R	CCTTAAAGTGGCTCACTTTACAAAGTAATGC<br>ATTCCCTTGGAACTAGGCCAGTTTAACTC	chr4:55547103-55547282
u99_mKLF4_GRE1_F<br>v1_mKLF4_GRE1_R	GCTAATGAGTCTAGAGCTGAGAGC<br>GGAGATAGATGGGGACCATCACTTTAT	chr4:55549100-55549271
p86_mFKBP5_p3472_F<br>p86a_mFKBP5_p3472_R	TGGACGGAATGTTCTAGTCAGGGCAG<br>ACCAGCCCAGGTTTAGCCTTGCT	chr17:28628146-28628238
p89_mFKBP5_p3469_F<br>p90_mFKBP5_p3469_R	CAGGCCAGCAGCATACAGGAAGA<br>GCATCGTCTGCGTCTTTCCCACAT	chr17:28600899-28601017
p91_mFKBP5_p3468_F	ACCTAGCTCAGCACAATGCTGGAC	chr17:28579402-28579534

p92_mFKBP5_p3468_R	ACCATTCCCTGCTGTTTAGGCGC	
p95_mFKBP5_p3465_F1<br>p96_mFKBP5_p3465_R2	GCACATCAAGTGAGTCTGGTCACTGC<br>TGCCAGCCACATTCAGAACAGGG	chr17:28557168-28557294
h27_rRNA_F<br>h28_rRNA_R	GATCCTTCGATGTCGGCTCTTCCTATC<br>AGGGTAAAACTAACCTGTCTCACG	GI:225637499
