# Supplementary material for: Glucocorticoid receptor coordinates transcription factor-dominated regulatory network in macrophages
Source: BMC Genomics. 2014 Aug 6;15(1):656. doi: 10.1186/1471-2164-15-656 (PMC4133603; doi:10.1186/1471-2164-15-656)
Supplement: Supplementary file 3 — Additional file 3: Figure S1: Is RT-qPCR confirmation of RNA-seq expression data for Dex-responsive genes encoding TFs. Figure S2. shows the effect of Chx on the basal and Dex-regulated levels of GR-responsive transcripts. Figure S3. shows the dynamics of Klf9 induction by Dex compared to the predicted expression of a putative Klf2 repressor in the GR-induced I1-FFL. Figure S4. shows ChIP-seq data for acute Dex-induced GR recruitment to genomic binding sites in mouse macrophages. Figure S5. demonstrates that GR binds to multiple sites within the Fkbp5 gene in response to Dex. (PDF 1 MB) [file 12864_2014_6351_MOESM3_ESM.pdf]

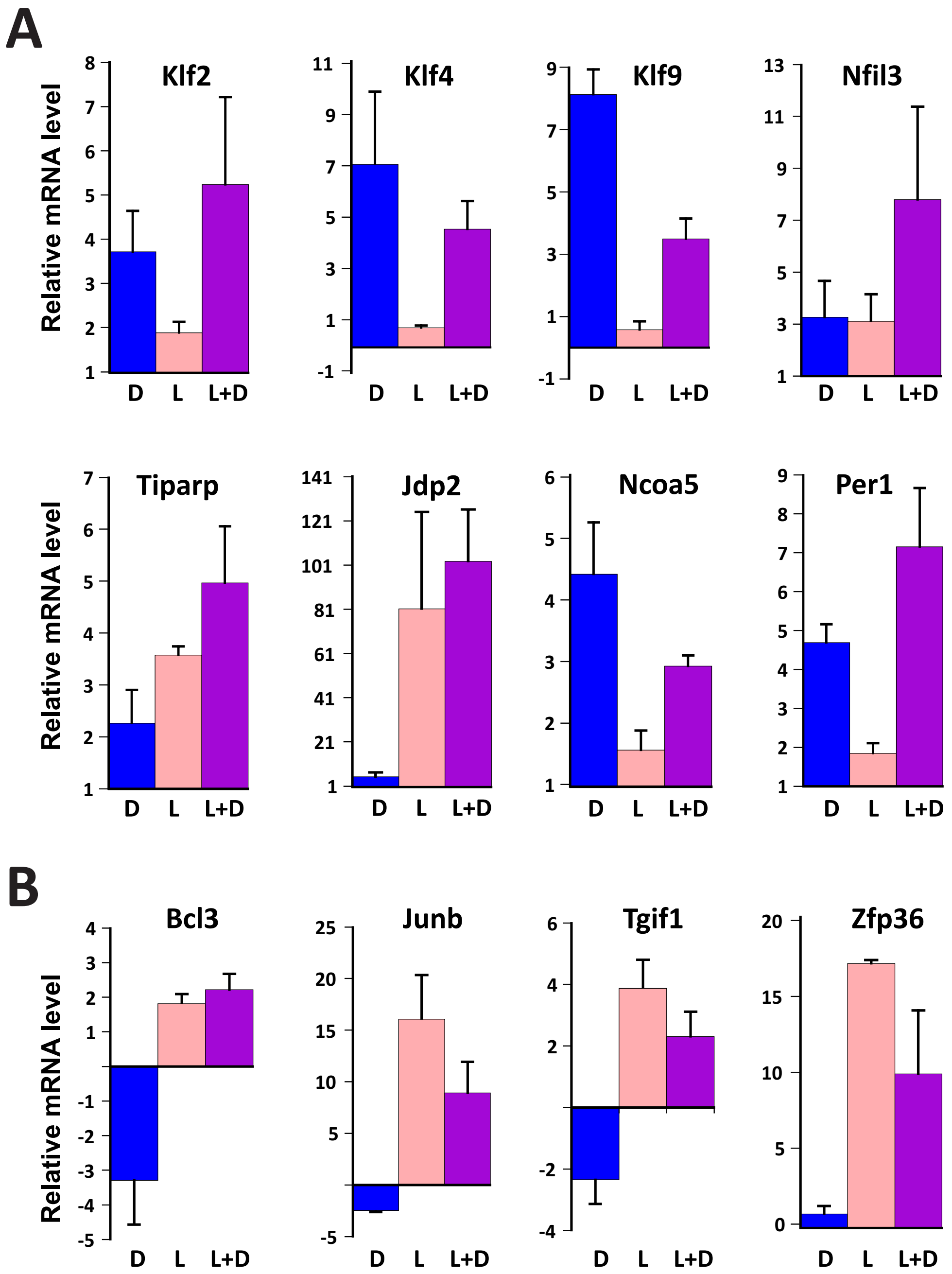
A
Relative mRNA level
Klf2
Klf4
Klf9
Nfil3
Tiparp
Jdp2
Ncoa5
Per1
D
L
L+D
B
Bcl3
Junb
Tgif1
Zfp36

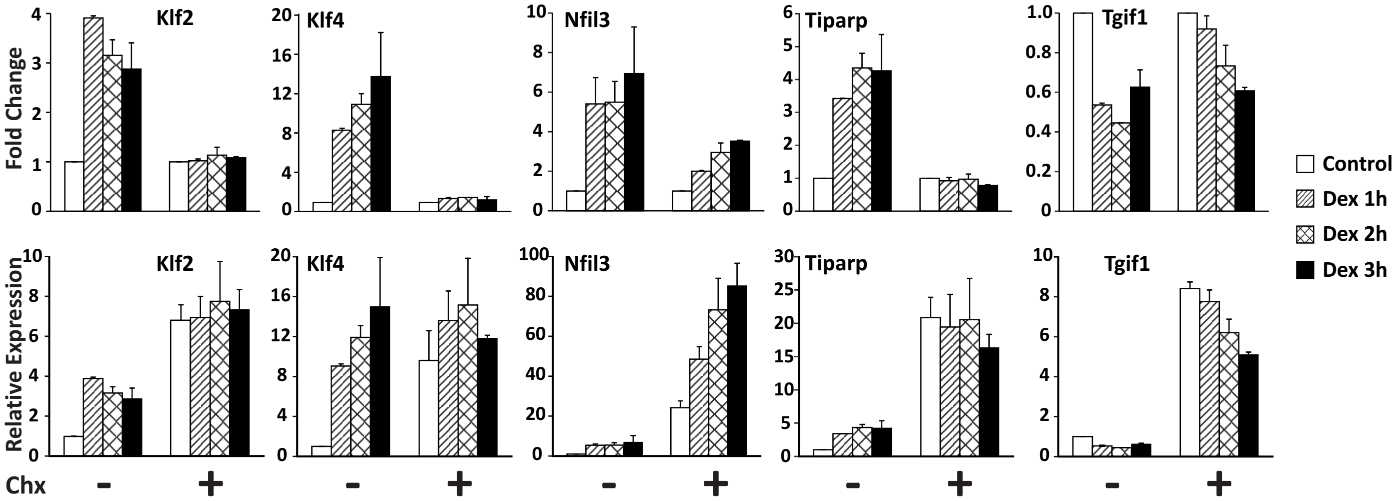

Fold Change
Relative Expression
Klf2
Klf4
Nfil3
Tiparp
Tgif1
Control
Dex 1h
Dex 2h
Dex 3h
Chx
-
+

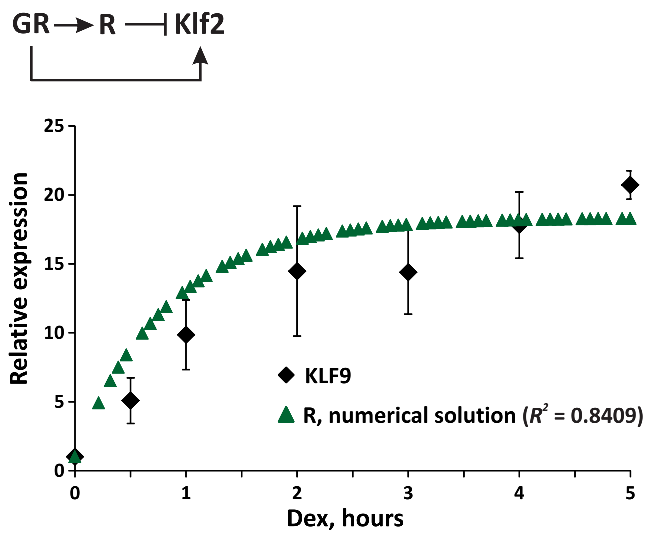

GR
R
Klf2
Relative expression
Dex, hours
KLF9
R, numerical solution (R^2 = 0.8409)
0
5
10
15
20
25
0
1
2
3
4
5

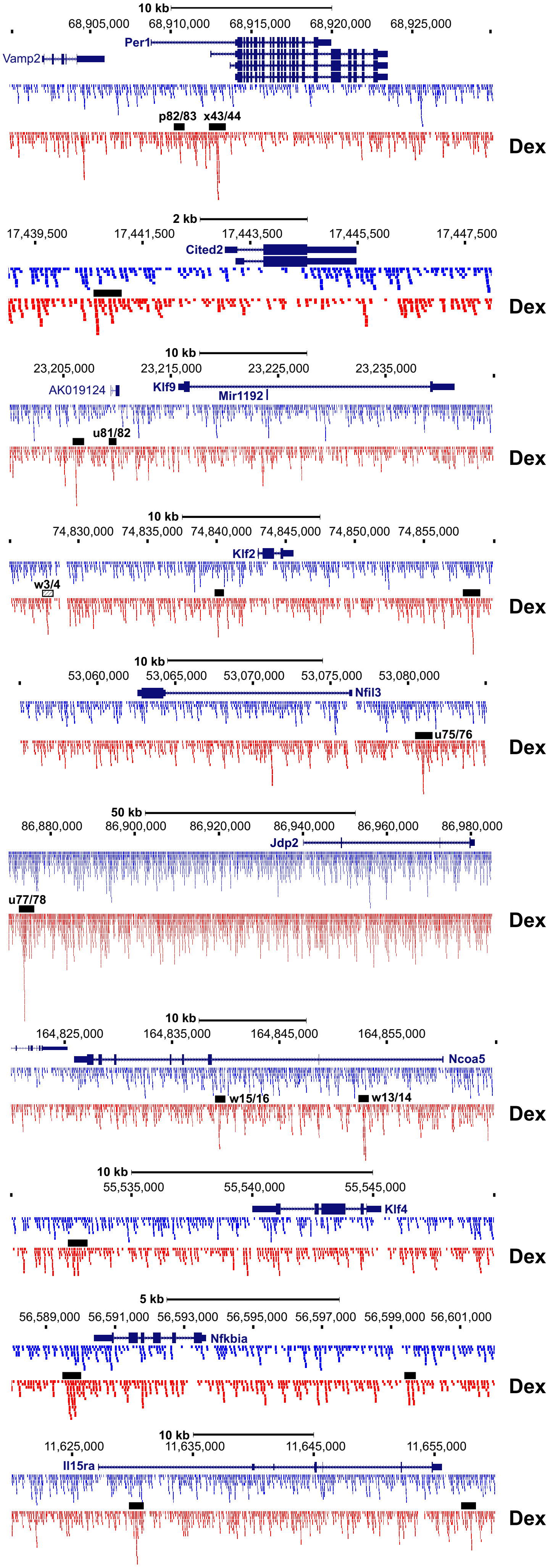
10 kb
68,905,000 68,910,000 68,915,000 68,920,000 68,925,000
Per1
Vamp2
p82/83 x43/44
Dex
2 kb
17,439,500 17,441,500 17,443,500 17,445,500 17,447,500
Cited2
Dex
10 kb
23,205,000 23,215,000 23,225,000 23,235,000
AK019124
Klf9
Mir1192
u81/82
Dex
10 kb
74,830,000 74,835,000 74,840,000 74,845,000 74,850,000 74,855,000
Klf2
w3/4
Dex
10 kb
53,060,000 53,065,000 53,070,000 53,075,000 53,080,000
Nfil3
u75/76
Dex
50 kb
86,880,000 86,900,000 86,920,000 86,940,000 86,960,000 86,980,000
Jdp2
u77/78
Dex
10 kb
164,825,000 164,835,000 164,845,000 164,855,000
Ncoa5
w15/16 w13/14
Dex
10 kb
55,535,000 55,540,000 55,545,000
Klf4
Dex
5 kb
56,589,000 56,591,000 56,593,000 56,595,000 56,597,000 56,599,000 56,601,000
Nfkbia
Dex
10 kb
11,625,000 11,635,000 11,645,000 11,655,000
Il15ra
Dex

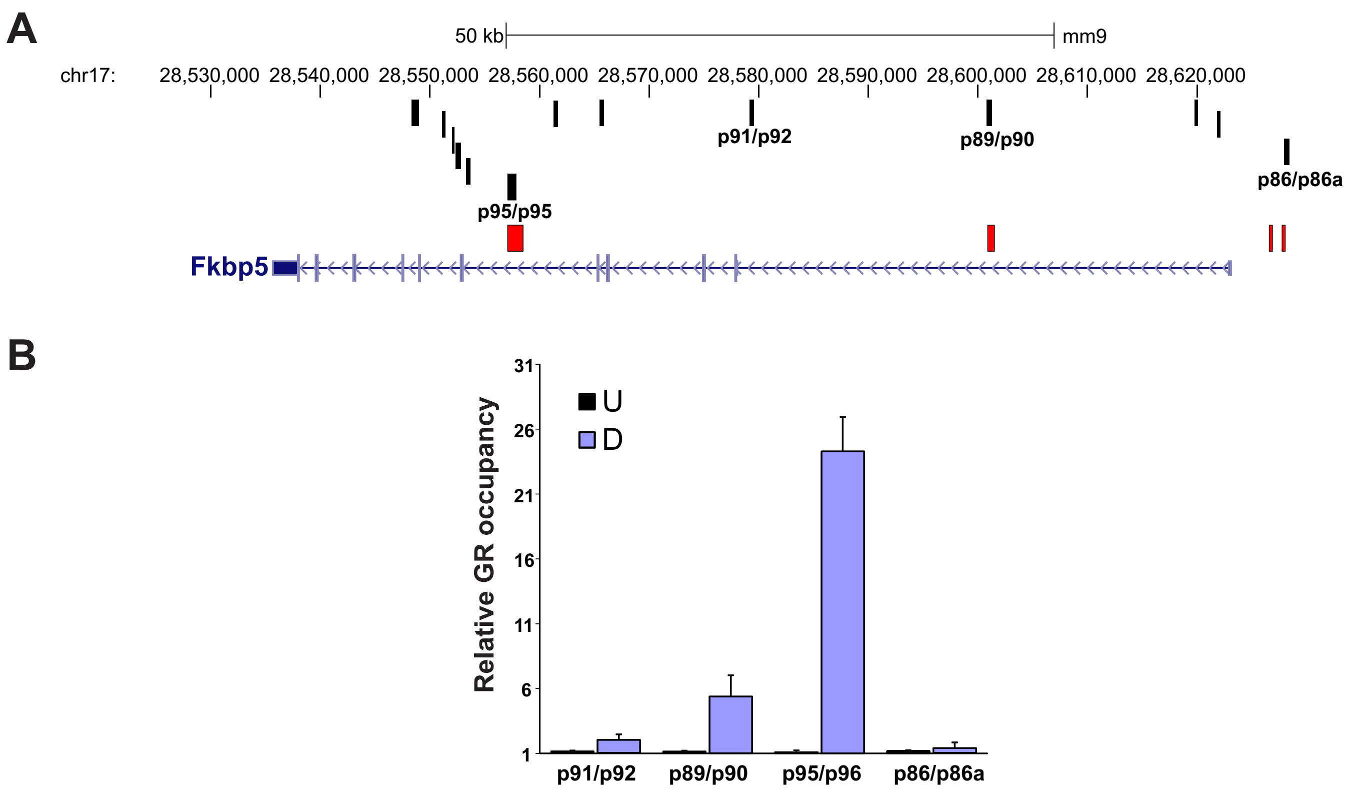
A
50 kb
mm9
chr17:
28,530,000
28,540,000
28,550,000
28,560,000
28,570,000
28,580,000
28,590,000
28,600,000
28,610,000
28,620,000
p91/p92
p89/p90
p86/p86a
p95/p95
Fkbp5
B
Relative GR occupancy
31
26
21
16
11
6
1
U
D
p91/p92
p89/p90
p95/p96
p86/p86a

Supplemental Figure Legends

Figure S1 RT-qPCR confirmation of RNA-seq expression data for Dex-responsive genes encoding TFs. BMMΦ were treated with Dex (D), LPS (L) or LPS+Dex (L+D) for 1 h, and transcript abundance for indicated (**A**) Dex-induced and (**B**) Dex-downregulated genes was assessed by RT-qPCR with Act1 mRNA as an internal control. The data are expressed relative to mRNA levels in untreated BMMΦ as fold over 1 for up- and fold over -1 for downregulated genes. Note that for Zfp36, the repressive effect of Dex is only detectible in LPS-treated BMMΦ. Shown is the average of 2 or 3 independent experiments, error bars are SD.

Figure S2 The effect of Chx on the basal and Dex-regulated levels of GR-responsive transcripts. BMMΦ were treated with Dex either alone or in the presence of the protein synthesis inhibitor Chx for 1-3 h; the expression of indicated genes were determined by RT-qPCR and expressed relative to transcript levels in the absence of Dex ('control') with or without Chx (top panel), or to 'control' without Chx only (bottom panel).

Figure S3 The dynamics of Klf9 induction by Dex compared to the predicted expression of a putative Klf2 repressor in the GR-induced I1-FFL. Klf9 expression data (black diamonds) collected over 5 h were compared to the kinetics of an intermediate repressor "R" (green triangles) in the I1-FFL regulating Klf2 expression (equation (3) in Additional file 3: Supplemental Methods). Expression data for Klf2 and Klf9 were derived from the same experiments. As Klf9 expression declines at later time points, only data representing a monotonous phase were used for the analysis. The quality of the fit has been evaluated by calculating the coefficient of determination R2, as described in Supplemental Methods (Additional file 3). The R2 for curve fitting analyses are shown in the plot legend.

Figure S4 Acute Dex-induced GR recruitment to genomic binding sites in mouse macrophages. BMMΦ were treated for 40 min -/+ Dex, as indicated, and genomic GR occupancy was assessed by ChIP-seq as described in Supplemental Methods (Additional file 3). A subset of Dex-induced GR binding peaks that were identified using CLC Bio Genomic Workbench (black bars) were individually validated by ChIP using pairs of primers listed in Additional file 1: Table S3.

Figure S5 GR binds to multiple sites within the Fkbp5 gene in response to Dex. (**A**) Chip-seq data from preadipocytes (black bars) and macrophages (red bars) revealed multiple GR binding peaks in the mouse Fkbp5 gene. The numbers under the putative GR binding sites indicate primer pairs used in ChIP experiments in MΦ (Additional file 1: Table S3). The gene annotation is according to the mm9 version of the mouse genome. (**B**) GR occupancy at putative GR binding sites indicated in (A) prior (U) and upon (D) a 1-h stimulation with 100 nM Dex as determined by ChIP.
